# Analysis and comparison of the trends in burden of spinal cord injury in China and worldwide from 1990 to 2021: an analysis of the global burden of disease study 2021

**DOI:** 10.3389/fpubh.2024.1517871

**Published:** 2025-01-07

**Authors:** Hao Qin, Yuhang Diao, Mingyu Hao, Zhitan Wang, Minghao Xie, Xiaojun Hu, Tao Zhu

**Affiliations:** ^1^Department of Neurosurgery, Tianjin Medical University General Hospital, Tianjin, China; ^2^Department of Otolaryngology, Tianjin Medical University General Hospital, Tianjin, China

**Keywords:** spinal cord injury, GBD 2021, epidemiology, public health, Joinpoint regression, age-period-cohort analysis, ARIMA model

## Abstract

**Background:**

Spinal cord injury (SCI) is a globally prevalent neurological condition, often resulting in motor, sensory, and autonomic dysfunctions that lead to permanent disability. However, outdated epidemiological data hinder the development and implementation of effective public health policies. This study aimed to examine and compare trends in the burden of spinal cord injury—specifically incidence, prevalence, and years lived with disability (YLD)—in China and worldwide from 1990 to 2021, and to project these trends over the next 15 years.

**Methods:**

This study analyzed the characteristics of the SCI burden in China and globally, examining changes in incidence, prevalence, and years lived with disability (YLD) using open data from the Global Burden of Disease database covering 1990 to 2021. Additionally, Joinpoint and age-period-cohort (APC) analyses were conducted to provide insights into the epidemiological characteristics of the SCI burden. The autoregressive integrated moving average (ARIMA) model was then applied to project SCI trends for the next 15 years.

**Results:**

In 2021, the prevalence and incidence of SCI in China reached 2,766,277 and 99,363 cases, respectively, marking increases of 63.27 and 43.27% since 1990. From 1990 to 2021, the age-standardized prevalence rate (ASPR) of SCI decreased significantly, with an estimated annual percentage change (EAPC) of −0.34 (95% CI: −0.60 to-0.07). At the gender level, SCI prevalence and incidence were higher in men than in women. Joinpoint analysis revealed a significant decrease in the APC of the age-standardized incidence rate (ASIR) from 1990 to 2011 (APC = −0.98, *p* < 0.05), followed by a notable increase from 2011 to 2021 (APC = 2.05, *p* < 0.05). For ASPR, a significant decrease occurred from 2001 to 2005 (APC = −4.80, *p* < 0.05), while subsequent periods showed an increasing trend, particularly between 2010 and 2018 (APC = 1.43, *p* < 0.05) and 2018–2021 (APC = 2.84, *p* < 0.05). In terms of age-standardized YLD rates (ASYR), China experienced an overall downward trend from 1990 to 2010 (APC = −0.56 for 1990–2001; −5.97 for 2001–2005; −1.01 for 2005–2010, *p* < 0.05), followed by a slight upward trend post-2010, with fluctuations from 2010 and 2018 (APC = 0.88) and 2018–2021 (APC = 2.49, *p* < 0.05). Age-period-cohort analysis showed that the risk of SCI incidence increased with age in China, though both period and cohort effects demonstrated a significant downward trend. Projections indicate that by 2036, the ASIR and ASPR for SCI in China will reach 6 and 146 cases per 100,000 people, respectively.

**Conclusion:**

The number and burden of SCI in China and globally have increased in the past. Among them, men and older people are more likely to develop SCI than women and younger people. Although the ASPR and ASIR for SCI are predicted to show a downward trend over the next 15 years, policy makers should continue to consider interventions to minimize the risk.

## Introduction

Spinal cord injury is typically caused by traumatic factors (such as traffic accidents, falls from heights, and acts of violence) or non-traumatic factors (such as inflammation, tumors, spinal cord hemorrhage or ischemia, infections, and degenerative spine diseases). These injuries often lead to severe, multisystemic and lasting effects, including motor and sensory dysfunction (1, 2), autonomic dysfunction (3), and psychological consequences (4).

The global incidence of SCI is estimated to be 10–83 cases per million people, with substantial regional variation influenced by socioeconomic status, healthcare access, and local conditions (5). In developed countries, SCI is primarily caused by traffic accidents and sports activities, while in developing countries, work-related accidents and violent behavior are the main causes (6). In 1990, the annual cost of medical management for spinal cord injuries in the United States was estimated at $4 billion, placing a substantial financial and social burden on patients’ families and society (7). Despite this impact, up-to-date data regarding the epidemiology, burden, and temporal trends of SCI remain limited.

This study examined the epidemiology and disease burden of SCI in China and globally using data from the GBD 2021 study. It aimed to provide a detailed overview of the incidence, prevalence, and burden of SCI. Additionally, the study projected the incidence and prevalence of SCI in China and worldwide over the next 15 years. These insights are expected to contribute to an enhanced understanding of the global burden of SCI.

## Methods

### Data sources

The SCI data analyzed in this study were sourced from GBD 2021, which offers updated epidemiological estimates on the burden of 371 diseases and injuries from 1990 to 2021, covering 21 GBD regions and 204 countries and territories. These data are publicly available on the Global Health Data Exchange (GHDE) platform (8, 9), with detailed information on data sources, methods, and statistical modeling provided in previous reports (10). SCI-related data were extracted for China and globally from 1990 to 2021. For age group selection, both the “all ages” and “age-standardized” groups were analyzed, along with the following specific age groups: under 5 years, 5–9 years, 10–14 years, 15–19 years, 20–24 years, 25–29 years, 30–34 years, 35–39 years, 40–44 years, 45–49 years, 50–54 years, 55–59 years, 60–64 years, 65–69 years, 70–74 years, 75–79 years, 80–84 years, 85–89 years, 90–94 years, and 95 years and above. The study incorporated gender categories of “Both,” “Female,” and “Male.” Additionally, seven risk factors contributing to the burden of SCI were identified: falls, road injuries, drowning, poisonings, self-harm, interpersonal violence, and foreign body-related incidents. Global population projection data were obtained from the GBD database for 2017 to 2,100 (11) to support the projection of future SCI trends.

### Statistical analysis

This study presents age-standardized incidence rates (ASIR), prevalence rates (ASPR), and years lived with disability (YLD) rates (ASYR) associated with spinal cord injury (SCI). The YLD metric quantifies the burden of SCI by calculating the total years patients live with disability due to SCI. A standardized global population from the Global Burden of Disease (GBD) database was used to determine ASR. The heterogeneity of these rates was assessed using 95% uncertainty intervals (UI) (12).

### Estimated annual percentage change (EAPC) and percentage change

This study used the estimated annual percentage change (EAPC) to assess dynamic trends in SCI prevalence, incidence, and years lived with disability (YLD) from 1990 to 2021. EAPC was calculated to describe the long-term trend in the age-standardized rate (ASR) of SCI burden by fitting the natural logarithm of the ASR to the calendar year using a regression model: *y* = *α* + *β*x + ɛ, where y = ln (rate), x = calendar year, and ɛ is the error term. Here, *β* indicates an upward or downward trend in ASR. EAPC and its 95% confidence interval (CI) were calculated as 100 × (exp(β)-1) (13).

Age-standardized indicators were considered to be trending upward if both the EAPC value and the lower limit of the 95% CI were above 0. A downward trend was indicated if both the EAPC value and the upper limit of the 95% CI were below 0. A constant trend was defined when the 95% CI for EAPC included 0. Statistical significance was set at a *p* value <0.05. Additionally, this study used percentage change to quantify changes in prevalence, incidence, and YLD cases from 1990 to 2021, calculated as: Percentage change = (2021 cases−1990 cases) / 1990 cases. All statistical analyses and visualizations were conducted using R statistical software (version 4.2.2).

### Joinpoint regression analysis

Joinpoint regression models are a series of linear statistical models used to evaluate trends in the disease burden induced by SCI over time. The average annual percentage change (AAPC) and corresponding 95% confidence intervals (CI) were calculated using Joinpoint software (version 4.9.1.0, National Cancer Institute, Rockville, MD, United States) to identify disease burden trends (14, 15). Log-transformed age-standardized indices can be fitted to a regression model as ln(y) = *α* + *β*x + ɛ, where y represents the age-standardized index and x represents the calendar year. AAPC is calculated as 100 × (exp(β)-1), with the 95% CI derived from the model. An age-standardized indicator is considered to show an increasing trend if the 95% CI for AAPC is >0, a decreasing trend if the 95% CI is <0, and a stable trend if the CI includes 0. *p* values less than 0.05 are considered statistically significant. Joinpoint regression analysis was performed using Joinpoint software.

### Age-period-cohort (APC) model

Age-period-cohort (APC) modeling was used to evaluate the effects of age, period, and cohort on health outcomes. The age effect captures differences in SCI prevalence across age groups due to age-related factors. The period effect reflects the influence of time-specific factors, such as advancements in diagnostic techniques, on SCI prevalence. The cohort effect represents changes in SCI prevalence across birth years, likely due to varying exposure to risk factors. The APC model is based on a Poisson distribution and can describe SCI trends across age, period, and cohort while adjusting for each. However, due to the linear relationship among age, period, and cohort, the intrinsic estimator (IE) method was integrated into the APC model by Yang et al. (16) to estimate the independent effects of these three dimensions. In the APC model with the IE approach, age-specific incidence rates were grouped into consecutive 5-year age intervals (0–4, 5–9, …, 95–99 years), 5-year periods from 1990 to 2021, and corresponding 5-year birth cohorts (1897–1901, 1902–1906, … 2012–2016, 2017–2021) to estimate the net effects of age, period, and cohort on SCI incidence and prevalence. APC model fitting was performed using the Epi package (version 2.46) in R (version 4.2.2). Additionally, StataMP software was used for analysis.

### ARIMA prediction

The ARIMA model was used to predict SCI prevalence and incidence for the next 15 years. ARIMA (AutoRegressive Integrated Moving Average) is a forecasting method for time series data that combines autoregressive (AR) and moving average (MA) components with differencing (d) to stabilize the series. The ARIMA model has three key parameters: ***p***, ***d***, and ***q***, where ***p*** denotes the order of the autoregressive term, ***d*** denotes the degree of differencing, and ***q*** denotes the order of the moving average term. At the core of ARIMA is the process of differencing, which converts a non-stationary series into a stationary one, allowing for more effective modeling. To assess the stability of the differenced series, we used autocorrelation (ACF) and partial autocorrelation (PACF) plots. The optimal model was selected based on the Akaike Information Criterion (AIC) values using the auto.arima() function. This function applied different ARIMA models to univariate time series data, with the best model identified based on provided constraints (17, 18). Model residuals were tested for normality using QQ plots, ACF, and PACF plots, while the Ljung-Box test was employed to check for serial correlation in the residuals. We fitted the ARIMA models using the ‘forecast,’ ‘tseries,’ and ‘ggplot2’ packages in R (version 4.2.2). The ARIMA model was successfully fitted.

## Results

### Incidence, prevalence and YLD of SCI in China and globally

As shown in Table 1, the incidence of SCI in China rose from 69,352 cases (95% CI: 54,774 - 88,357) in 1990 to 99,363 cases (95% CI: 72,456 - 136,733) in 2021, reflecting a cumulative increase of 43.27%. The age-standardized incidence rate (ASIR) for SCI was 6.07 (95% CI: 4.76–7.84) per 100,000 in 1990 and 6.21 (95% CI: 4.65–8.40) per 100,000 in 2021, with an estimated annual percentage change (EAPC) of-0.22 (95% CI: −0.52 to 0.07). Globally, SCI incidence grew from 473,666 cases (95% CI: 377,726 - 598,764) in 1990 to 574,502 cases (95% CI: 440,219 - 757,445) in 2021, a cumulative rise of 21.29%. However, ASIR declined from 9.16 (95% CI: 7.28–11.68) per 100,000 in 1990 to 7.12 (95% CI: 5.48–9.36) per 100,000 in 2021, with an EAPC of-0.81 (95% CI: −0.93 to-0.69). Both in China and globally, SCI occurring at the neck level slightly exceeded SCI below the neck level in terms of prevalence.

**Table 1:** All-age cases and age-standardized prevalence, incidence, and YLDs rates in 1990 and 2021 for SCI in china and global.

Location	Cause	Measure	1990		2021		1990–2021 EAPC
			All-ages cases	Age-standardized rates per 100,000 people	All-ages cases	Age-standardized rates per 100,000 people	
			*n* (95% CI)	*n* (95% CI)	*n* (95% CI)	*n* (95% CI)	*n* (95% CI)
China	Spinal Injuries	Incidence	69,352 (54774–88,357)	6.07 (4.76–7.84)	99,363 (72456–136,733)	6.21 (4.65–8.4)	-0.22(−0.52–0.07)
		Prevalence	1,694,264 (1574087–1,843,869)	149.84 (139.72–162.12)	2,766,277 (2557986–3,007,581)	151.69 (140.39–164.98)	-0.34(−0.60 - -0.07)
		YLDs	594,359 (417490–760,095)	52.09 (36.86–66.42)	751,837 (533035–983,599)	41.61 (29.28–54.36)	-1.20(−1.49 - -0.90)
	Spinal cord lesion at neck level	Incidence	35,399 (25982–48,628)	3.12 (2.28–4.37)	52,454 (34148–81,575)	3.23 (2.16–4.94)	−0.14(−0.40–0.13)
		Prevalence	785,139 (730492–849,161)	68.95 (64.37–74.34)	1,315,929 (1216024–1,427,664)	72.16 (66.82–78.28)	−0.17(−0.41–0.06)
		YLDs	342,208 (243084–432,987)	29.84 (21.47–37.58)	499,615 (357658–648,198)	27.65 (19.59–35.92)	−0.63(−0.88 - -0.37)
	Spinal cord lesion below neck level	Incidence	33,953 (24481–47,595)	2.95 (2.09–4.35)	46,910 (29417–73,988)	2.98 (1.92–4.55)	−0.32(−0.64–0.01)
		Prevalence	909,125 (840773–992,282)	80.89 (75.14–87.77)	1,450,348 (1335882–1,589,808)	79.53 (73.18–87.26)	−0.48(−0.77 - -0.19)
		YLDs	252,152 (174994–329,317)	22.25 (15.51–28.99)	252,222 (175708–345,195)	13.96 (9.71–19.24)	−2.14(−2.50 - -1.79)
Global	Spinal Injuries	Incidence	473,666 (377726–598,764)	9.16 (7.28–11.68)	574,502 (440219–757,445)	7.12 (5.48–9.36)	−0.81(−0.93 - -0.69)
		Prevalence	10,820,146 (9937398–11,841,568)	222.7 (205.58–241.62)	15,400,682 (14009114–17,075,106)	183.56 (166.96–203.7)	−0.73(−0.77 - -0.69)
		YLDs	3,532,432 (2494013–4,600,912)	71.78 (50.99–93.01)	4,566,237 (3195694–6,007,036)	54.62 (38.2–71.97)	−1.00(−1.04 - -0.95)
	Spinal cord lesion at neck level	Incidence	249,122 (183942–345,205)	4.88 (3.56–6.88)	2,905,920 (2070218–3,764,134)	3.78 (2.69–5.6)	−0.83(−0.93 - -0.72)
		Prevalence	5,212,979 (4765776–5,759,968)	107.05 (98.39–116.87)	306,568 (216989–456,717)	88.47 (80.41–99.9)	−0.70(−0.74 - -0.67)
		YLDs	2,152,418 (1539248–2,733,084)	43.8 (31.45–55.38)	7,423,601 (6744305–8,352,639)	34.72 (24.77–44.95)	−0.84(−0.88 - −0.80)
	Spinal cord lesion below neck level	Incidence	224,545 (161480–306,922)	4.28 (3.06–5.88)	267,935 (181475–387,802)	3.33 (2.28–4.78)	-0.80(−0.93 - -0.66)
		Prevalence	5,607,168 (5110755–6,244,369)	115.65 (106.28–127.7)	7,977,081 (7150375–9,155,049)	95.09 (85.18–109.25)	−0.76(−0.80 - -0.71)
		YLDs	1,380,014 (970519–1,892,804)	27.99 (19.73–38.53)	1,660,317 (1148393–2,345,733)	19.89 (13.75–28.15)	−1.26(−1.31 - -1.20)

In China, the prevalence of SCI in 2021 reached 2,766,277 cases (95% CI: 2,557,986 - 3,007,581), a 63.27% increase from 1990. This corresponds to an age-standardized prevalence rate (ASPR) of 149.84 (95% CI: 139.72–162.12) per 100,000 in 1990, compared to 151.69 (95% CI: 140.39–164.98) per 100,000 in 2021, with an EAPC of-0.34 (95% CI: −0.60 to-0.07). Globally, the number of SCI cases rose from 10,820,146 (95% CI: 9,937,398 - 11,841,568) in 1990 to 15,400,682 (95% CI: 14,009,114 - 17,075,106) in 2021. However, ASPR decreased from 222.70 (95% CI: 205.58–241.62) per 100,000 in 1990 to 183.56 (95% CI: 166.96–203.70) per 100,000 in 2021, with an EAPC of-0.73 (95% CI: −0.77 to-0.69) (Table 1).

For years lived with disability (YLD), China reported 751,837 cases (95% CI: 533,035 - 983,599) in 2021, compared with 594,359 cases (95% CI: 417,490 - 760,095) in 1990, marking a 26.50% increase. Nevertheless, the ASYR declined from 52.09 (95% CI: 36.86–66.42) per 100,000 in 1990 to 41.61 (95% CI: 29.28–54.36) per 100,000 in 2021, with an EAPC of-1.20 (95% CI: −1.49 to-0.90). Globally, YLDs also increased, yet ASYR decreased from 71.78 (95% CI: 50.99–93.01) per 100,000 in 1990 to 54.62 (95% CI: 38.20–71.97) in 2021, with an EAPC of-1.00 (95% CI: −1.04 to-0.95) (Table 1).

In addition, in the analysis of the incidence, prevalence, and YLDs of SCI across 21 GBD regions (Supplementary Table S1), distinct trends were observed between 1990 and 2021. In most regions, there was an increasing trend in the number of cases, prevalence, and YLDs. Conversely, in Western Europe, Eastern Europe, Central Europe, and high-income Asia-Pacific regions, both the number of SCI cases and the age-standardized rates showed a declining trend. This decline aligns with the findings reported in the EAPC report.

### Trends in SCI disease burden in China and globally

**Figure 1:**
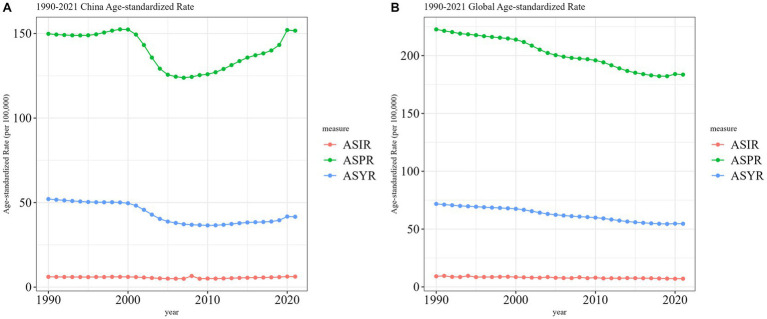
Trend comparison of ASIR, ASPR, and ASYR of SCI in China and worldwide from 1990 to 2021. **(A)** China; **(B)** Worldwide.

As shown in Figure 1, the ASIR of SCI in China and globally remained generally stable from 1990 to 2021, although China experienced a notable increase from 2017 to 2018, surpassing the global trend. From 1990 to 2010, China’s ASYR for SCI showed a decreasing trend, with a more pronounced decline from 2000 to 2008. Between 2011 and 2020, ASYR exhibited a minor upward trend, though it remained lower overall compared to 1990 levels. Globally, ASYR demonstrated a consistent downward trend throughout the period. In China, the ASPR exhibited an initially stable and rapid decline followed by a slow increase, with significant changes primarily between 2000 and 2005. In contrast, the global ASPR consistently showed a downward trend.

**Figure 2:**
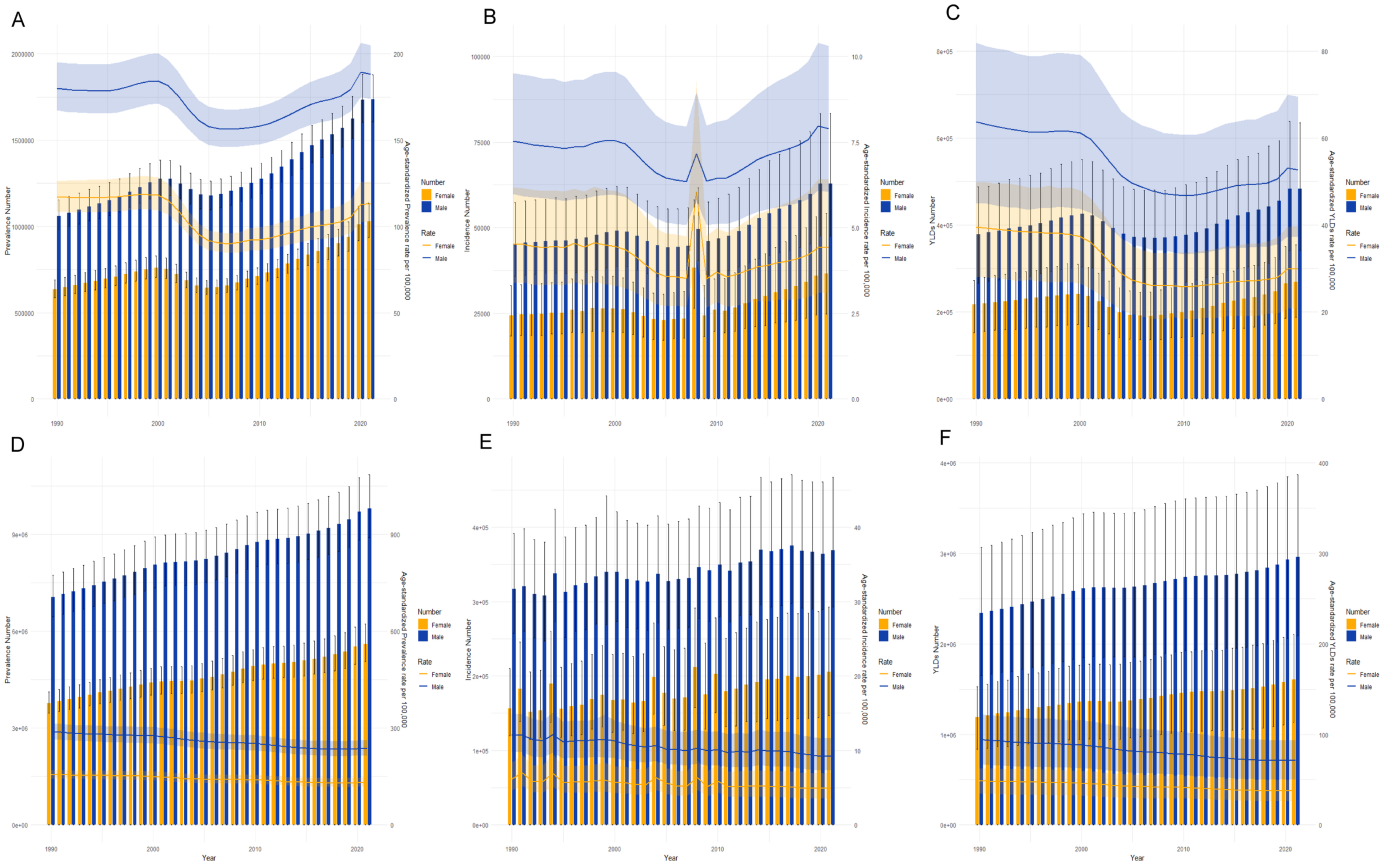
Trends in the all-age cases and age-standardized prevalence, incidence, and YLDs rates of SCI by sex from 1990 to 2021 in china and global. **(A)** prevalence number and rate of SCI in china. **(B)** Incidence number and rate of SCI in china. **(C)** YLD number and rate of SCI in china. **(D)** Prevalence number and rate of SCI in global. **(E)** Incidence number and rate of SCI in global. **(F)** YLD number and rate of SCI in global.

Figure 2 illustrates sex-specific trends in all-age numbers and age-standardized rates of SCI incidence, prevalence, and YLD in China and worldwide from 1990 to 2021. In China, both the incidence and prevalence of SCI showed an overall upward trend. However, aside from a small peak in the ASIR in 2008 (Figure 2B), the ASPR and ASYR exhibited fluctuations over the years (Figures 2A,C). Notably, the ASYR exhibited an overall downward trend (Figure 2C), a pattern consistent across both sexes, although the incidence among males was significantly higher than among females. Globally, age-standardized incidence, prevalence, and YLD rates declined for both males and females, despite a continued increase in the total number of SCIs (Figures 2D–F).

In addition, Supplementary Figure S1 depicts trends in ASPR and ASIR across different genders and regions categorized by varying social demographic indices (SDI) over the years. Our analysis indicates that high SDI regions consistently showed higher ASPR values for both males and females. Despite this, ASPR generally demonstrated a downward trend in middle-high SDI regions, high SDI regions, and globally. In contrast, ASPR remained relatively stable in middle, middle-low, and low SDI regions. The trend in ASIR mirrored that of ASPR overall; however, ASIR exhibited marked fluctuations in low SDI regions. Notably, for both genders, ASIR experienced sharp increases in 1994 and 2010, surpassing the rates observed in high SDI regions.

### Joinpoint analysis of SCI burden in China and globally

**Figure 3:**
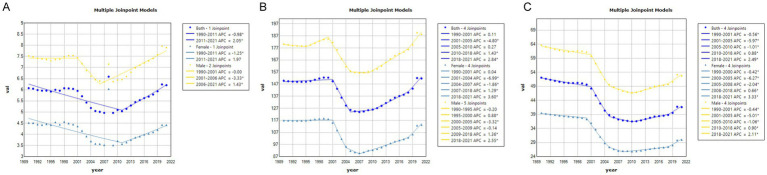
The APC of ASIR, ASPR, and ASYR of SCI in China from 1990 to 2021 (* means *p*-values<0.05 and significant results). **(A)** ASIR; **(B)** ASPR; **(C)** ASYR.

**Figure 4:**
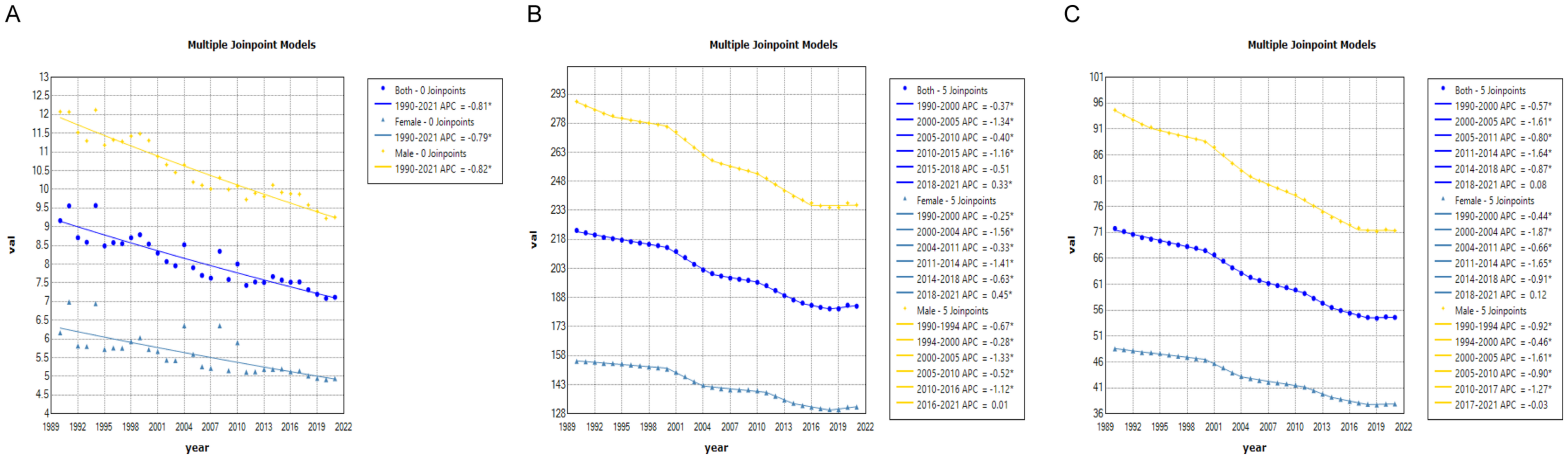
The APC of ASIR, ASPR, and ASYR of SCI in Global from 1990 to 2021 (* means *p*-values<0.05 and significant results). **(A)** ASIR; **(B)** ASPR; **(C)** ASYR.

Figures 3, 4 depict the Joinpoint regression trends of ASIR, ASPR, and ASYR for SCI in China and globally. In China, the APC of ASIR for SCI showed a significant decrease from 1990 to 2011 (APC = −0.98, *p* < 0.05), followed by a significant increase from 2011 to 2021 (APC = 2.05, *p* < 0.05) (Figure 3A and Supplementary Table S2). Globally, however, the ASIR APC for SCI consistently decreased over the entire period from 1990 to 2021 (APC = −0.81, *p* < 0.05) (Figure 4A and Supplementary Table S5). For ASPR, China demonstrated a significant decrease in APC from 2001 to 2005 (APC = −4.80, *p* < 0.05), followed by an upward trend in subsequent periods, particularly from 2010 to 2018 (APC = 1.43, *p* < 0.05) and 2018 to 2021 (APC = 2.84, *p* < 0.05) (Figure 3B and Supplementary Table S3). In contrast, globally, ASPR showed a consistent and significant decrease (*p* < 0.05) (Figure 4B and Supplementary Table S6). Regarding ASYR, China’s APC demonstrated a significant decline during multiple periods (1990–2001 APC = −0.56; 2001–2005 APC = −5.97; 2005–2010 APC = −1.01, *p* < 0.05) (Figure 3C and Supplementary Table S4). Although a similar downward trend was observed globally (1990–2000 APC = −0.57; 2000–2005 APC = −1.61; 2005–2011 APC = −0.80; 2011–2014 APC = −1.64; 2014–2018 APC = −0.87, *p* < 0.05) (Figure 4C and Supplementary Table S7), China exhibited a moderate upward trend post-2010, with APC values of 0.88 from 2010 to 2018 and 2.49 from 2018 to 2021 (*p* < 0.05), indicating some volatility (Figure 3C).

### Gender differences in SCI burden in different age groups in China and globally

**Figure 5:**
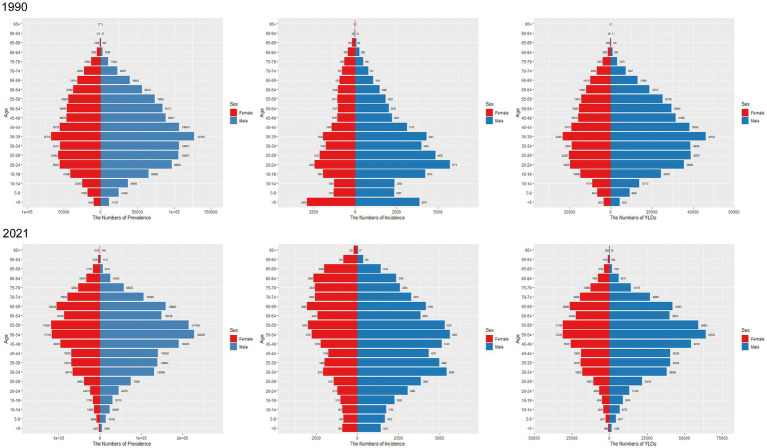
Comparison of the number of prevalence, incidence, and YLDs of SCI in males and females of different age groups in China in 1990 and 2021.

Figure 5 illustrates the incidence, prevalence, and YLD trends for SCI across different age groups for men and women in China in 1990 and 2021. In terms of incidence, the incidence was higher among males than females. In 1990, the peak incidence for males was in the 20–24 age group, while for females it was in the under-5 group. Across all age groups up to 75 years, males had higher SCI incidence than females, whereas after 75, the incidence was higher in females. By 2021, the peak incidence for males shifted to the 30–34 and 50–54 age groups, and for females to the 55–59 and 65–69 groups, with the incidence being higher among women than men after age 80.

**Figure 6:**
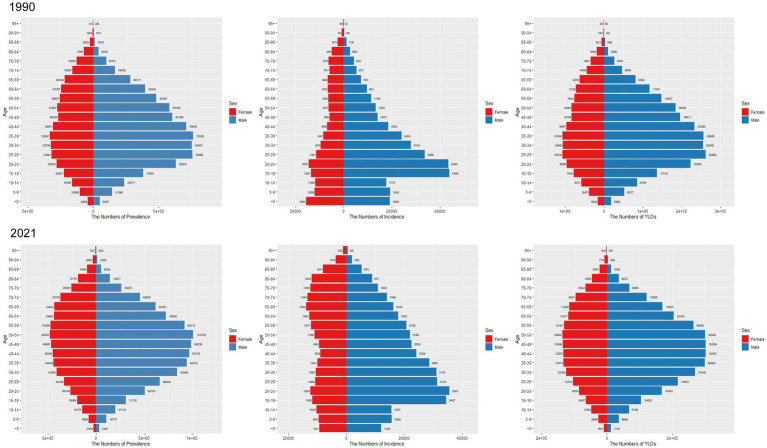
Comparison of the number of prevalence, incidence, and YLDs of SCI in males and females of different age groups in worldwide in 1990 and 2021.

Globally, in 1990, the highest incidence for males was observed in the 15–19 age group, while for females it was observed in the under-5 group. Males had a higher incidence than females up to 70 years of age, after which the incidence was higher among females (Figure 6). In 2021, peak incidence for males was in the 20–24 age group, while incidence among females exhibited greater variability (Figure 6).

Regarding prevalence, in 1990, the number of SCI cases in both sexes increased from the under-5 group to the 35–39 group, peaking in the 35–39 group, and then declined with age, mirroring the trend observed in China (Figure 5). In 2021, the peak prevalence in China was observed in the 50–54 age group for males and the 55–59 group for females (Figure 5). Globally, high prevalence was concentrated in the 35–39, 40–44, 45–49, 50–54, and 55–59 age groups, with prevalence rising with age until 54 and then decreasing (Figure 6).

Regarding YLD, in China, YLD for both sexes peaked in the 35–39 age group in 1990. After age 70, YLD were greater in females compared to males (Figure 5). By 2021, YLD peaked in the 50–54 group, and once again, YLD were greater for females compared to males after age 80 (Figure 5). Globally, YLD in 1990 showed a smoother distribution in the 25–29, 30–34, and 35–39 groups (Figure 6). By 2021, this trend had shifted to the 35–39, 40–44, 45–49, and 50–54 groups (Figure 6). In both years, YLD were consistently greater in females compared to males after age 80.

### Impact of age, period, and cohort on incidence and prevalence

**Figure 7:**
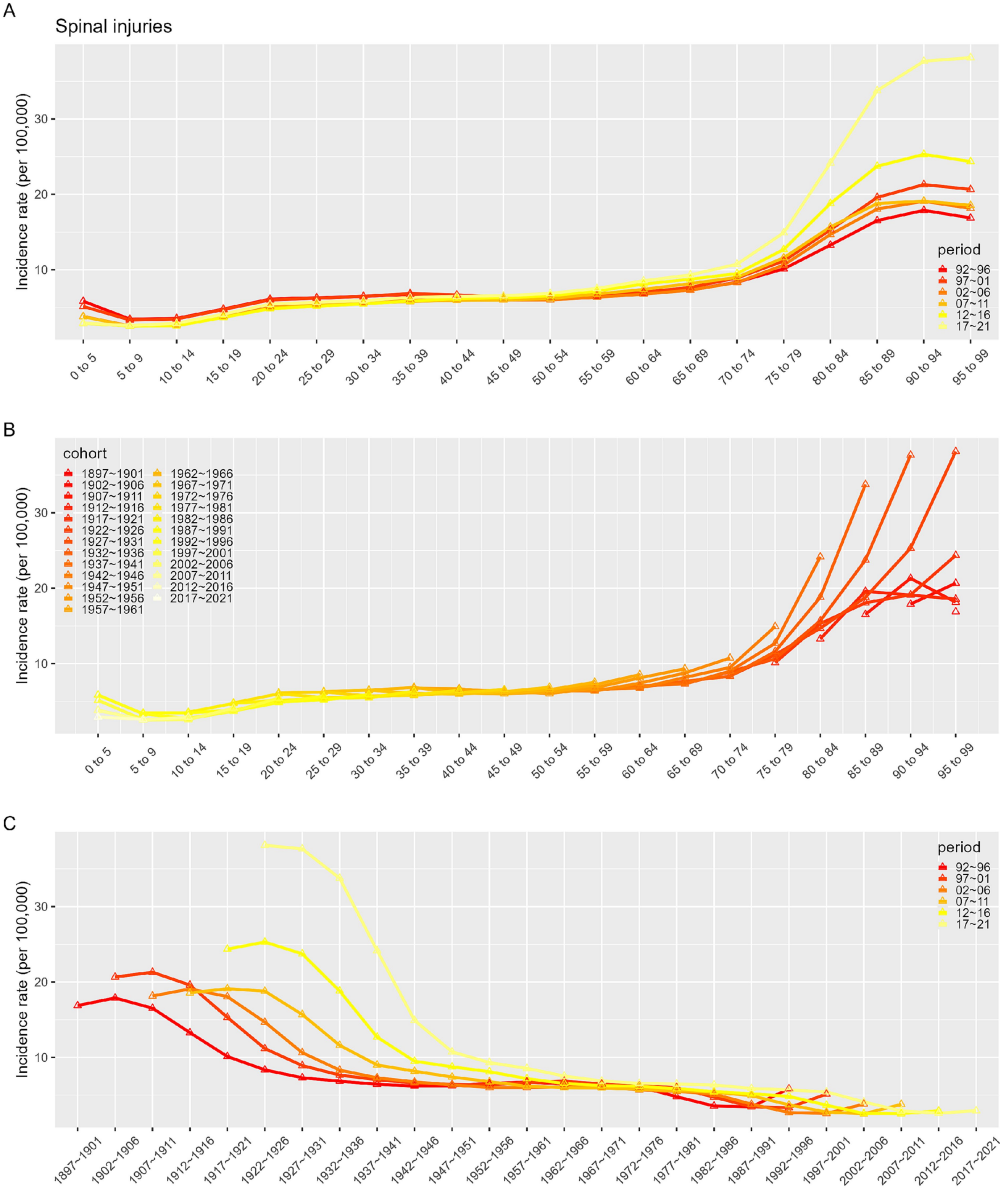
Incidence rates of SCI in China. **(A)** The age-specific incidences rates of SCI according to time periods; each line connects the age-specific incidence for a 5-year period. **(B)** The age-specific incidences rates of SCI according to birth cohort; each line connects the age-specific incidence for a 5-year cohort. **(C)** The birth cohort-specific incidence rates of SCI according to time periods; each line connects the birth cohort-specific incidence for a 5-year period.

**Figure 8:**
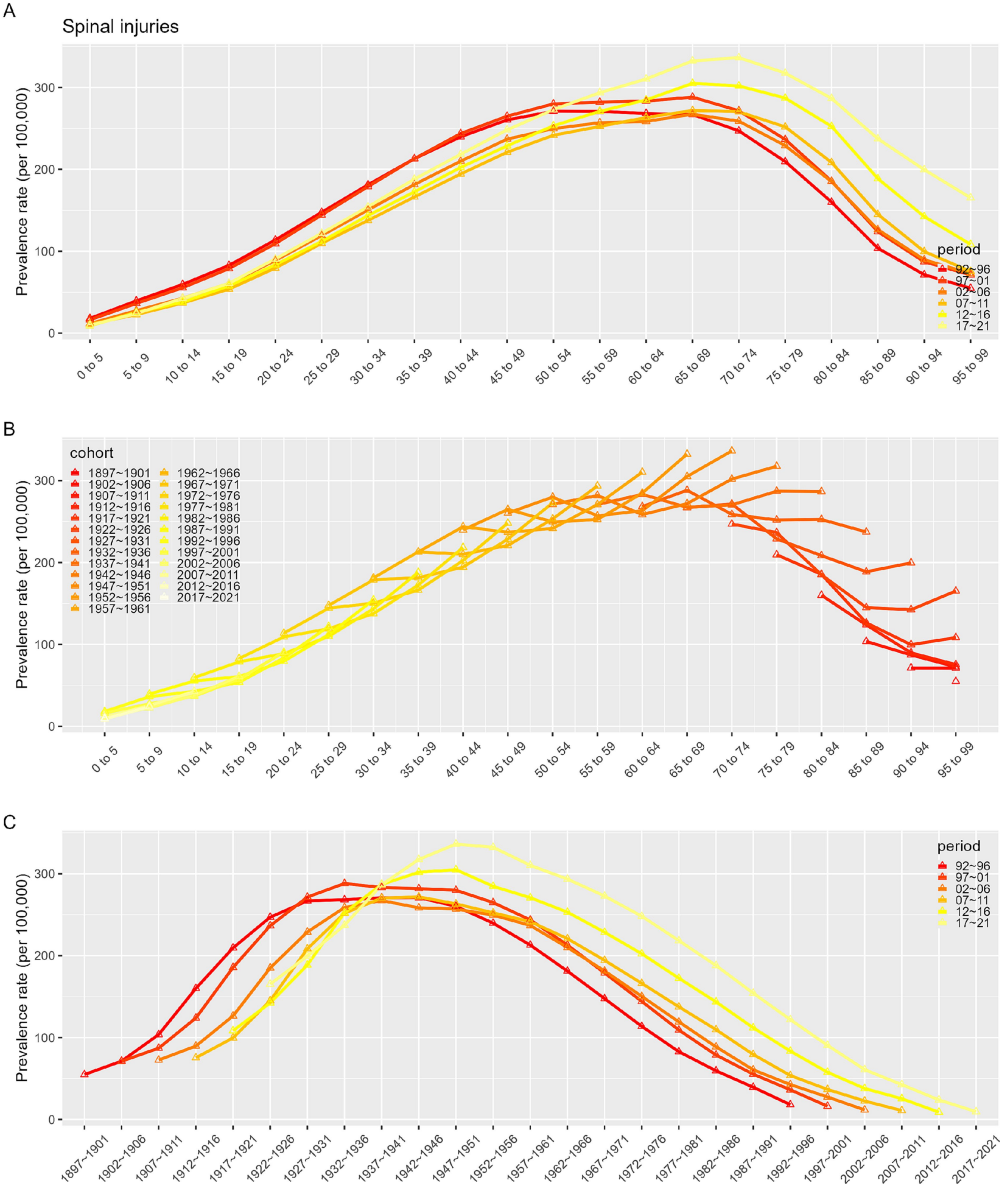
Prevalence rates of SCI in China. **(A)** The age-specific incidences rates of SCI according to time periods; each line connects the age-specific incidence for a 5-year period. **(B)** The age-specific incidences rates of SCI according to birth cohort; each line connects the age-specific incidence for a 5-year cohort. **(C)** The birth cohort-specific incidence rates of SCI according to time periods; each line connects the birth cohort-specific incidence for a 5-year period.

**Figure 9:**
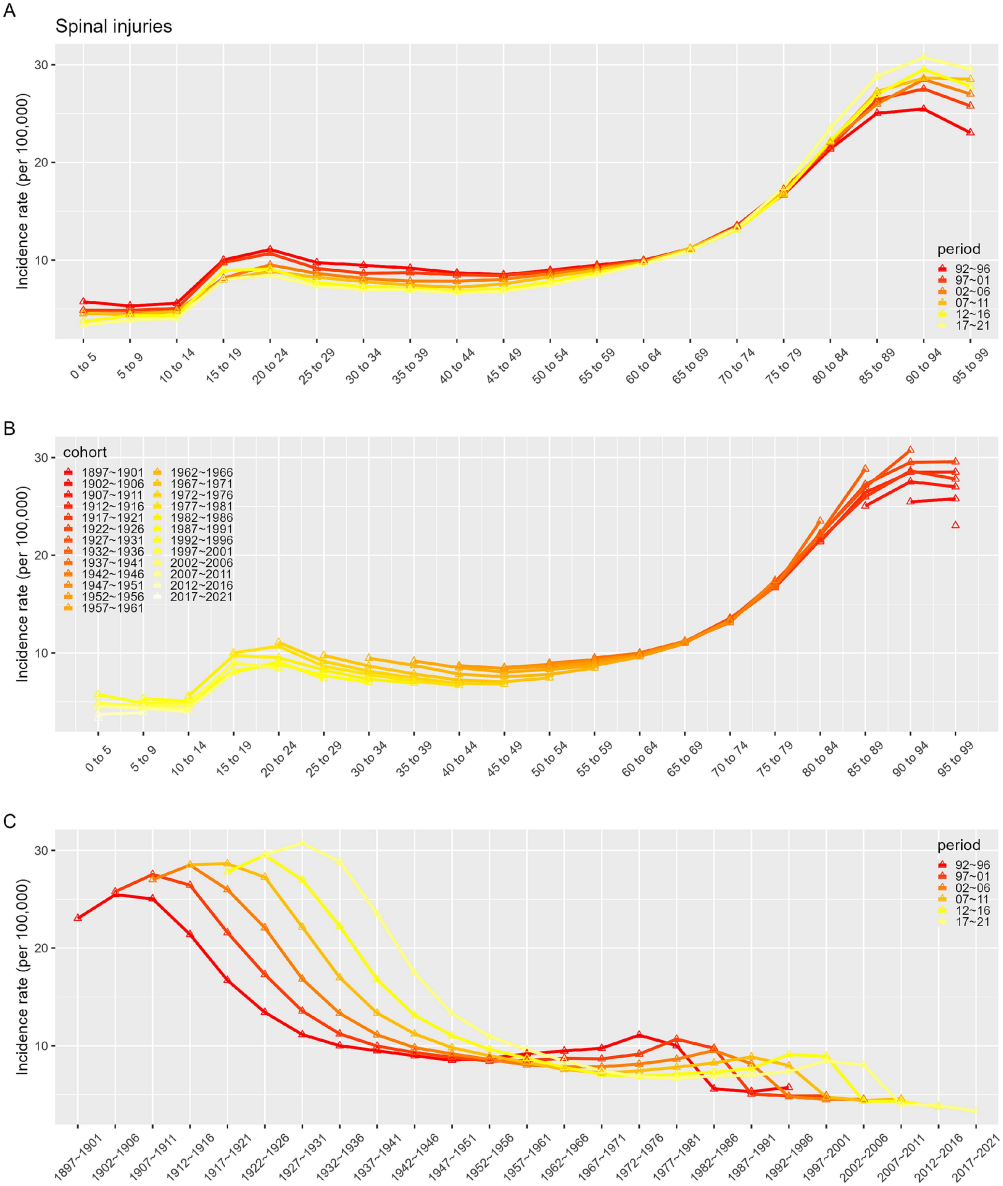
Incidence rates of SCI in Global. **(A)** The age-specific incidences rates of SCI according to time periods; each line connects the age-specific incidence for a 5-year period. **(B)** The age-specific incidences rates of SCI according to birth cohort; each line connects the age-specific incidence for a 5-year cohort. **(C)** The birth cohort-specific incidence rates of SCI according to time periods; each line connects the birth cohort-specific incidence for a 5-year period.

**Figure 10:**
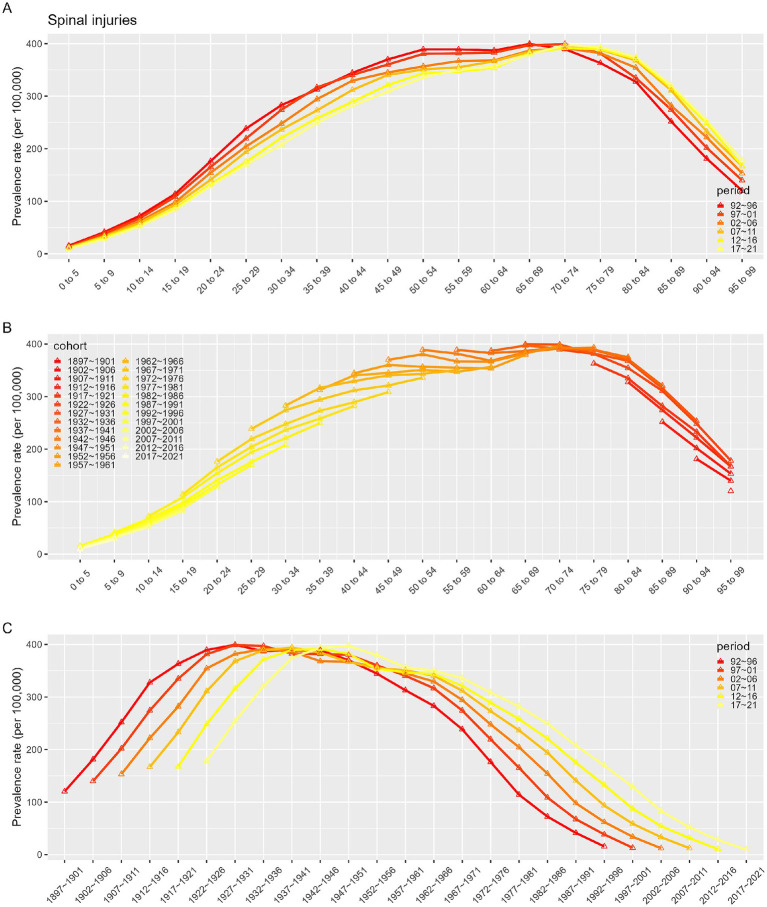
Prevalence rates of SCI in Global. **(A)** The age-specific incidences rates of SCI according to time periods; each line connects the age-specific incidence for a 5-year period. **(B)** The age-specific incidences rates of SCI according to birth cohort; each line connects the age-specific incidence for a 5-year cohort. **(C)** The birth cohort-specific incidence rates of SCI according to time periods; each line connects the birth cohort-specific incidence for a 5-year period.

Figures 7–10 depict the incidence and prevalence of SCI in China and globally across different age groups, cohorts, and time periods. Both in China and worldwide, SCI incidence rates generally increase with age. In China, incidence rose sharply after age 70 (Figure 7A), while globally, there were notable increases after ages 10–19 and 60 (Figure 9A). Despite these age-related trends, SCI incidence rates in both China and globally exhibited an overall downward trend over time (Figures 7B, 9B). In particular, China saw a significant decline in incidence prior to the 1947–1951 period (Figure 7C). In contrast to incidence rates, SCI prevalence in China and globally demonstrated an initial increase followed by a decrease with age (Figures 8, 10). In both regions, peak prevalence was in the 65–69 age group before 2012; after 2012, this peak shifted to the 70–74 age group. Similarly, SCI prevalence in China and globally exhibited a rising trend that later declined over time. Detailed information can be found in Supplementary Tables S8–S11.

### ARIMA

**Figure 11:**
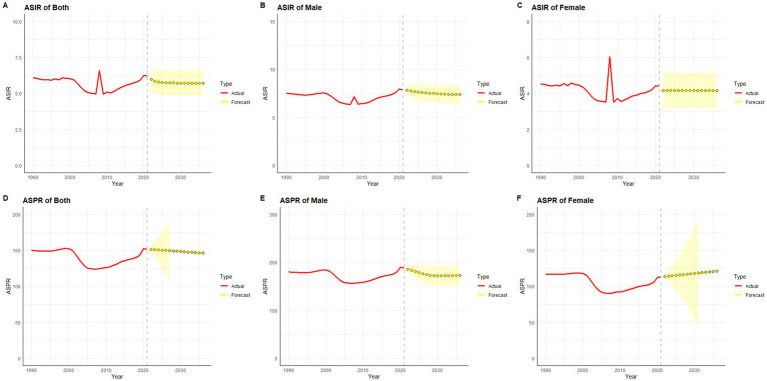
Predicted trends of SCI ASIR and ASPR in China over 15 years (2022–2036). The red lines represent the true trend of age–standardized incidence and prevalence of SCI during 1990–2021; the yellow lines represent the predicted trend and the light–yellow shaded regions represent the 95% confidence interval of predicted values; the gray dot vertical line split data into true value (1990–2021) and predicted value [2022–2036]. **(A)** ASIR of both; **(B)** ASIR of male; **(C)** ASIR of female; **(D)** ASPR of both; **(E)** ASPR of male; **(F)** ASPR of female.

**Figure 12:**
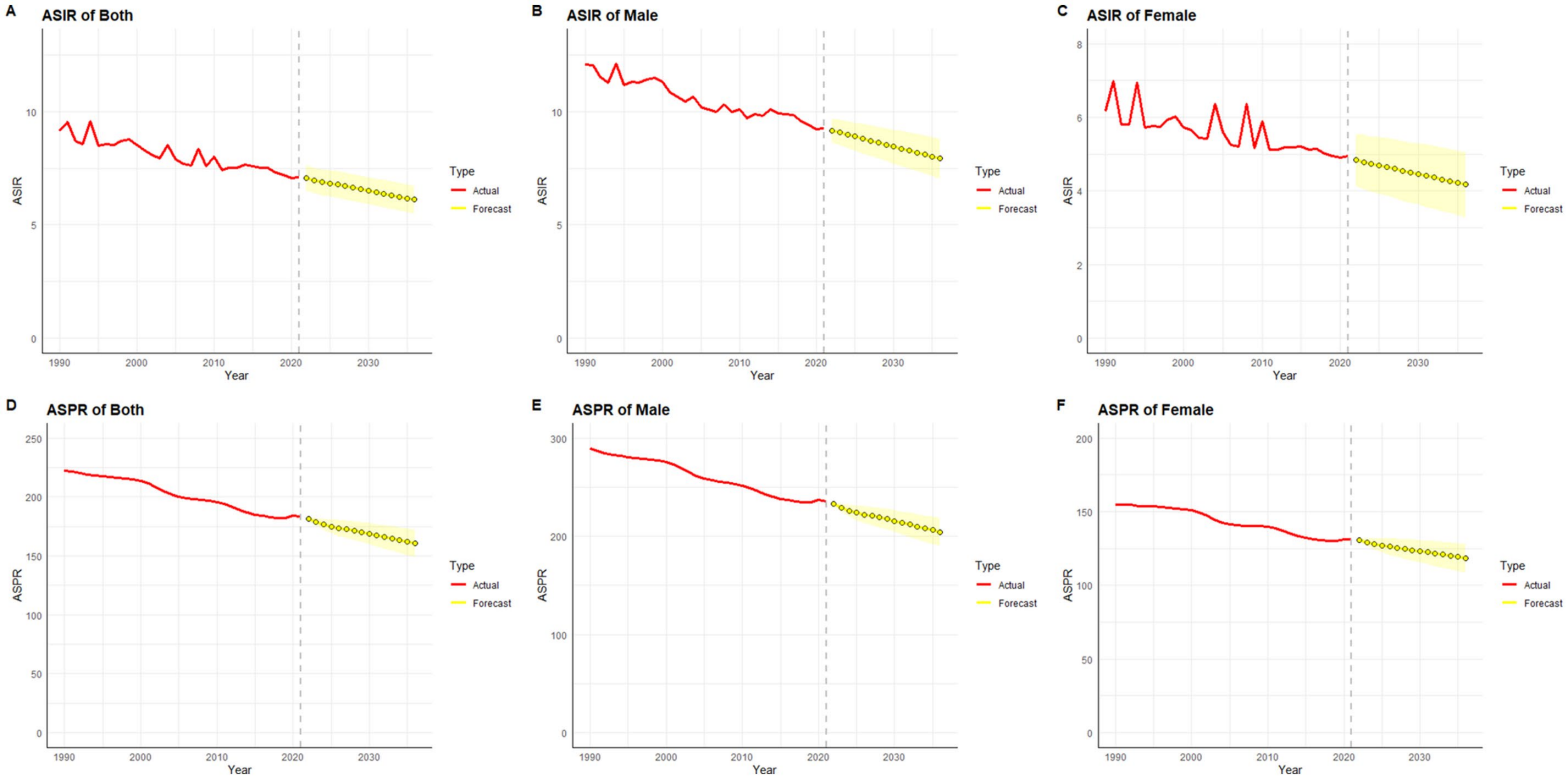
Predicted trends of SCI ASIR and ASPR in Global over 15 years (2022–2036). The red lines represent the true trend of age–standardized incidence and prevalence of SCI during 1990–2021; the yellow lines represent the predicted trend and the light–yellow shaded regions represent the 95% confidence interval of predicted values; the gray dot vertical line split data into true value (1990–2021) and predicted value [2022–2036]. **(A)** ASIR of both; **(B)** ASIR of male; **(C)** ASIR of female; **(D)** ASPR of both; **(E)** ASPR of male; **(F)** ASPR of female.

Using SCI incidence and prevalence data from 1990 to 2021, we employed an ARIMA model to forecast trends for the next 15 years. Overall, both ASIR and ASPR of SCI in China and globally are predicted to decline over this period (Figures 11, 12).

In gender-stratified predictions for Chinese ASIR, the ARIMA model parameters selected were (1,0,0) for males and (0,0,0) for females, identified using the auto.arima() function, with AICs of 6.1 and 49.84, respectively. Q-Q plots, ACF, and PACF plots confirmed the normality of residuals (Supplementary Figure S2), and the Ljung-Box test validated the robustness of the model, with residuals exhibiting white noise (*p*-values of 0.83 and 0.84, respectively). ASIR in men is projected to decrease over the next 15 years (Figure 11B), while ASIR in women is expected to remain relatively stable (Figure 11C). For global SCI incidence, ASIR for both genders is projected to decline (Figures 12B,C), with optimal ARIMA models with parameters (0,1,0) for males and (5,1,0) for females, yielding AICs of 10.69 and 34.83, respectively. Q-Q plots, ACF, and PACF plots validated the normality of residuals (Supplementary Figure S3), and the Ljung-Box test confirmed model stability (*p*-values of 0.99 and 0.32, respectively).

For ASPR predictions, China’s ASPR in males is projected to decline initially and then stabilize, while in females, it is projected to gradually increase over the next 15 years (Figures 11E,F). The ARIMA models used were (2,0,0) for males and (0,2,0) for females, with AICs of 154.52 and 125.95, respectively. Q-Q plots, ACF, and PACF plots validated the normality of residuals (Supplementary Figure S4), and the Ljung-Box tests confirmed the robustness of the model, with residuals displaying white noise (p-values of 0.99 and 0.94, respectively). Globally, ASPR for both males and females is projected to decrease over the next 15 years (Figures 12E,F). The optimal ARIMA parameters were (2,1,0) for both genders, with AICs of 83.83 and 53.68, respectively. Q-Q plots, ACF, and PACF plots validated the normality of residuals (Supplementary Figure S5), and the Ljung-Box test confirmed the robustness of the model (p-values of 0.85 and 0.90, respectively).

### Etiology analysis results for SCI

The analysis of the causes of SCI burden in China and globally for the years 1990 and 2021 is depicted in Supplementary Figure S6. In 1990, drowning and foreign body-related incidents were identified as the primary causes of SCI globally and in China. By 2021, the global SCI burden remained predominantly attributed to foreign body-related incidents. However, in China, self-harm had emerged as the leading cause of SCI burden. Furthermore, from 1990 to 2021, there was a slight decrease in the proportion of SCIs resulting from falls, road injuries, and interpersonal violence, while the proportion attributable to poisonings.

## Discussion

This study comprehensively assessed the incidence, prevalence, and YLD of SCI in China and globally, based on data from the GBD 2021 database. Differences in the burden of SCIs across ages and genders in China and worldwide were compared, trends were analyzed using the Joinpoint and APC models, and the ARIMA model was applied to forecast SCI incidence and prevalence for the next 15 years. The findings revealed a significant increase in the prevalence, incidence, and YLD of SCI in China and globally from 1990 to 2021. Although the absolute number of people affected by SCI has increased with population growth, the age-standardized incidence and prevalence rates remained relatively stable. This stability can be attributed to several factors, including enhancements in traffic safety regulations, improvements in emergency medical services, and advancements in rehabilitation technologies (19). A decline in China’s ASPR of SCI was observed between 2000 and 2005, in contrast to the global trend. This decline can be attributed to several historical events in China during this period. Notably, China’s accession to the World Trade Organization (WTO) in 2001 marked a significant milestone in the nation’s economic reform and opening-up, which spurred rapid economic growth, improved infrastructure, and enhanced healthcare conditions. These developments led to an increase in light labor occupations, thereby reducing the prevalence of SCI. Additionally, the SARS epidemic outbreak in 2003 likely curtailed work and travel activities, resulting in a substantial decrease in occupational and traffic accidents, further contributing to the reduction in SCI prevalence. However, an upward trend in China’s ASIR was observed in 2008, possibly associated with the severe Sichuan earthquake that year. Post-2008, China’s ASPR exhibited an increasing trend, which can be attributed to factors such as an aging population, the continued expansion of the industrial and construction sectors, and heightened traffic density. Furthermore, advancements in medical technology, particularly in imaging and neurosurgical techniques, have significantly improved the diagnostic capabilities for spinal cord injuries, influencing the observed trends. In addition, in the analysis of SCI burden across 21 GBD regions, only Europe and high-income Asia-Pacific regions were found to have experienced a reduction in SCI burden. This can be attributed to the advanced healthcare systems, comprehensive preventive measures, and efficient rehabilitation services commonly found in these regions (20). These findings suggest that higher levels of economic development are typically associated with more effective SCI prevention and treatment strategies.

In our etiological analysis, an increase was observed in the percentage of SCI attributed to self-harm in China in 2021. This rise can be attributed to a combination of psychological and social factors. The intense pressure from societal competition and daily life can contribute to the development of psychological issues, such as depression, in vulnerable individuals. In severe instances, these psychological problems may manifest as self-injurious behaviors, subsequently leading to SCI. Furthermore, the widespread use of the Internet has led to increased exposure to cyber-violence for some individuals, resulting in psychological trauma that, in extreme cases, can also trigger self-harm. Conversely, both in China and globally, a slight decrease was observed in the proportion of SCIs caused by falls, road injuries, and interpersonal violence in 2021. This decline may be attributed to cultural advancements, enhanced traffic policing, and improvements in personal conduct. Additionally, with the growth of the aging population, greater attention is being given to the older adult, with heightened efforts in areas such as fall prevention, which may contribute to this trend.

In both China and globally, ASIR and YLD were greater for neck-level SCI than below-neck injuries, whereas ASPR was higher for below-neck injuries. Neck-level SCIs represent about 50% of traumatic SCIs and are more prevalent than thoracolumbar injuries (21). Traumatic SCIs can lead to multi-organ dysfunction, especially affecting the cardiovascular and respiratory systems (21). In children, approximately 80% of SCI occur at the neck level, whereas only 30% occur in adults, which can be attributed to children’s larger head size relative to their body and underdeveloped paraspinal musculature (22, 23). Additionally, prolonged labor and abnormal newborn positioning during delivery contribute to an increased risk of SCI in children.

Regarding age and gender, older adults and males exhibit higher prevalence of SCI compared to younger adults and females. Older adults are particularly susceptible to falls due to physical and health declines, with increased life expectancy linked to a higher risk of conditions such as spinal stenosis, osteoporosis, and fall-related injuries, which elevate SCI risk (24). Men generally have a higher prevalence of SCI, likely due to greater involvement in physical labor and high-risk activities, whereas women, often engaged in lower-energy or family-based activities, are less exposed to injury risks (25). Studies also show that male children have a higher propensity for SCI compared to female children, and that adolescents are more susceptible to SCI compared to preschoolers (26).

Given the complex interactions of age, period, and cohort factors, APC modeling with the intrinsic estimator (IE) was applied to quantify the net effects of these factors. The APC analysis revealed that the age effect on SCI incidence consistently increased over time, while SCI prevalence demonstrated an initial increase followed by a decline with age. It was observed that between 1990 and 2021, while the incidence in younger populations remained stable, the incidence in older populations exhibited an increasing trend across various time points. As individuals age, reduced mobility and muscle activity can lead to significant bone density loss, heightening the risk of falls, fractures, and SCI (27, 28). Aging is also associated with degenerative spinal conditions like stenosis and disc herniation, which contribute to SCI risk (24). Therefore, prevention, early identification, and treatment of spinal cord injuries in the older adult population are particularly important. Period effects showed a trend of decline in incidence but an initial increase followed by a decrease in prevalence, potentially reflecting changes in living standards, healthcare, and social conditions. The cohort effect, representing early-life socioeconomic, behavioral, and environmental influences, showed a peak in SCI incidence, which gradually declined thereafter, while prevalence initially increased, peaked for the 1947–1951 cohort before declining. This pattern likely reflects socioeconomic and medical advances over time, with especially high effects before 1947–1951, when social instability, poor nutrition, and limited healthcare access were prevalent. In more recent generations, increased health awareness and improved living standards have promoted chronic disease prevention.

When analyzing the changes in ASPR and ASIR across different SDI regions over the years, it was observed that low SDI regions exhibited significant fluctuations in ASIR. This may be attributed to factors such as underdeveloped transportation infrastructure, limited medical resources, unstable socioeconomic conditions, and frequent wars and conflicts. This trend suggests that in the early stages of social development, these uncertain factors may lead to an increased burden of SCI (29). Therefore, low SDI regions should draw lessons from high SDI regions and take proactive measures to reduce future SCI risks (30).

This study provides updated insights into the burden of SCI in China and globally from 1990 to 2021, potentially aiding policymakers in comprehending current epidemiological trends. However, this study has several limitations. First, as the SCI data source is based on patient medical records, the reported estimates may underestimate the actual incidence and prevalence of SCI. Second, ASPR was calculated using the GBD standard population rather than the Chinese population, which, while facilitating international comparison, may not fully capture the prevalence given China’s large aging population. Third, the lack of provincial-level data in China precluded geographic analysis of SCI burden and ARIMA projections at the provincial level. Lastly, as the APC model was analyzed at the population level, results could be influenced by ecological fallacy.

## Conclusion

As the global population has grown, the incidence, prevalence, and YLDs of SCI have risen in both China and globally, while ASIR, ASPR, and ASYR have declined during 1990–2021. Men and older adults are disproportionately affected by SCI compared to women and younger individuals. In terms of disability impact, neck-level SCIs have the most severe consequences. With an aging global population, preventing falls and managing degenerative spinal conditions in the older adult has become a critical priority. Although SCI prevalence and incidence are projected to decline over the next 15 years, ongoing advancements in prevention and management strategies are essential.

## Data Availability

The datasets presented in this study can be found in online repositories. The names of the repository/repositories and accession number(s) can be found in the article/Supplementary material.
