# Supplementary material for: Analysis and comparison of the trends in burden of spinal cord injury in China and worldwide from 1990 to 2021: an analysis of the global burden of disease study 2021
Source: Front Public Health. 2025 Jan 7;12:1517871. doi: 10.3389/fpubh.2024.1517871 (PMC11747465; doi:10.3389/fpubh.2024.1517871)
Supplement: Supplementary file 1 [file Data_Sheet_1.pdf]

Supplementary Table 1. All-age cases and age-standardized prevalence, incidence, and YLDs rates in 1990 and 2021 for SCI in 21 GBD regions.

Location	Measure	Number_1990(95%CI)	Number_2021(95%CI)	ASR_1990(95%CI)	ASR_2021(95%CI)	EAPC(95%CI)
Andean Latin America	Prevalence	52895 (45613-65624.5)	100819.3 (91407.1-114038.8)	159.5 (141.9-189.8)	153.3 (138.9-173.7)	-0.25 (-0.36 to -0.13)
	Incidence	3153.9 (2324.1-4413.9)	3370.4 (2670.3-4248.3)	7.9 (5.9-10.8)	5.1 (4-6.4)	-0.9 (-1.22 to -0.59)
	YLDs	19575 (13997.2-26921.5)	30924.3 (22040.2-40665.2)	58.3 (42-78.1)	46.9 (33.3-61.6)	-0.82 (-0.93 to -0.71)
Australasia	Prevalence	109745.7 (99257.5-121169.6)	155482.4 (139903.6-174153.9)	503 (455.3-555.2)	417.6 (371.6-467.6)	-0.58 (-0.68 to -0.48)
	Incidence	3505.7 (2731.9-4461.4)	4769 (3464.1-6695.4)	17.1 (13.4-21.8)	14 (10.5-18.8)	-0.55 (-0.62 to -0.49)
	YLDs	30315.6 (21306.1-39990.7)	42591.7 (30189.6-55503.5)	139.4 (97.7-184.5)	116.1 (81.3-151.9)	-0.57 (-0.67 to -0.47)
Caribbean	Prevalence	42651.2 (39887.1-45815.5)	98805.8 (81292.6-126878.4)	133.2 (125.2-142.7)	198.9 (162-257.9)	1.77 (1.41 to 2.12)
	Incidence	1911.3 (1560-2355.9)	3509.5 (2758.9-4514.7)	5.6 (4.5-6.9)	7.3 (5.7-9.3)	0.76 (-1.02 to 2.57)
	YLDs	14470.9 (10211.7-18438.8)	32636.4 (22038.3-47573.5)	44.7 (31.6-57)	66.2 (44.5-97.7)	1.85 (1.4 to 2.3)
Central Asia	Prevalence	134004.9 (123195.9-147251.9)	174855.5 (159192.8-193329.4)	217.9 (200.7-238.5)	178.6 (162.5-197.5)	-0.73 (-0.79 to -0.68)
	Incidence	5610.4 (4516.9-7051.8)	5985.2 (4809.5-7467.9)	8 (6.4-10.1)	6.2 (5-7.8)	-1.41 (-2.04 to -0.77)
	YLDs	45613.7 (32800.3-58612.3)	54946.5 (38179.5-72501)	73.5 (53-94.4)	55.9 (38.9-73.7)	-1.02 (-1.09 to -0.94)
Central Europe	Prevalence	455492.6 (418325.5-497429.6)	423456.2 (385656.9-466755.9)	334.6 (307-365.2)	286.9 (260.8-317.8)	-0.55 (-0.66 to -0.44)
	Incidence	18170.1 (13859.5-23760.9)	14138.1 (10176.9-19849.4)	14.2 (10.9-18.5)	10.8 (8.1-14.3)	-1.42 (-1.62 to -1.22)
	YLDs	140154.9 (100656.6-180743.2)	111405.8 (78114.7-146847.1)	103.6 (74.3-133.7)	77.1 (53.2-102.5)	-1.02 (-1.1 to -0.93)
Central Latin America	Prevalence	389662.4 (341966.9-467569)	529858.3 (483520.2-589758.6)	277 (247.6-319.2)	200.5 (183.1-223)	-0.6 (-0.82 to -0.38)
	Incidence	16802.7 (13359.7-21457.7)	17823.6 (14027.4-22716.3)	10.4 (8.2-13.3)	7 (5.5-8.9)	-0.97 (-1.2 to -0.74)
	YLDs	138862.3 (99245.6-186824.3)	163107.6 (116056.7-215266.9)	97.2 (70.2-128.6)	61.7 (43.9-81.2)	-1.01 (-1.22 to -0.8)
Central Sub-Saharan Africa	Prevalence	48356.2 (38686.6-65470.4)	135153.5 (102382.6-189283.1)	103.9 (86.5-134.2)	127.7 (96.9-179.4)	0.61 (0.23 to 0.99)
	Incidence	2861.4 (2167.8-3843.2)	5933 (4700.2-7642)	5.3 (4.1-7)	4.7 (3.7-6)	-2.04 (-3.43 to -0.62)
	YLDs	19249.6 (12895.4-27866.9)	49485.4 (32915.9-73667.2)	40.6 (27.8-57.6)	46 (30.5-68.4)	0.33 (-0.08 to 0.74)
East Asia	Prevalence	1747560.2 (1624462-1899308.5)	2828676.4 (2618044.2-3072196.7)	149.3 (139.3-161.3)	149.8 (138.7-162.8)	-0.36 (-0.61 to -0.1)
	Incidence	71649.1 (56609.5-91187)	101621.6 (74344.8-139538.5)	6.1 (4.7-7.8)	6.1 (4.6-8.3)	-0.26 (-0.55 to 0.03)
	YLDs	611600.4 (430100.7-782566.1)	770023.8 (546247.6-1007151.9)	51.8 (36.6-66)	41.2 (29-53.8)	-1.2 (-1.49 to -0.92)
Eastern Europe	Prevalence	931556 (856699.7-1017258.7)	767900.2 (702227-837709.9)	372 (341.8-407.1)	296.7 (271.4-325.3)	-0.8 (-0.94 to -0.65)
	Incidence	35496.6 (27723-45628.8)	26392.7 (20167.4-35168.1)	15.4 (12.1-19.6)	12.2 (9.5-16)	-1.14 (-1.45 to -0.82)
	YLDs	283129.6 (204084.7-366906.1)	206895 (145611.7-267059.3)	113.9 (82.1-147.5)	81.3 (56.6-105.8)	-1.21 (-1.39 to -1.02)
Eastern Sub-Saharan Africa	Prevalence	221309.9 (149629.6-352690.4)	423525.4 (316307.2-622487.4)	131.1 (95.1-198.4)	131.6 (97.1-196.2)	-0.19 (-0.48 to 0.09)
	Incidence	29273 (16217.7-51831)	18551 (13863.2-25919.7)	14.1 (8.3-24.6)	4.6 (3.6-6.4)	-3.12 (-4.49 to -1.74)
	YLDs	88236 (53969.1-145179.7)	154213.5 (100099.3-241121.2)	51.4 (33.2-81.9)	47 (30.3-74.2)	-0.51 (-0.82 to -0.21)
High-income Asia Pacific	Prevalence	718288.8 (663593-780901.5)	653587.8 (603162.9-716763)	368.6 (340.5-400.8)	250.6 (231.1-276.2)	-1.44 (-1.54 to -1.33)
	Incidence	24730.3 (19618.6-31638.7)	19157.6 (14175-26546.1)	13.8 (10.9-17.6)	8.6 (6.6-11.3)	-1.75 (-1.87 to -1.64)
	YLDs	211626.6 (151424-274487.4)	179536.1 (128533.7-235013)	109 (78.1-141.5)	70.4 (49.8-92.4)	-1.58 (-1.65 to -1.51)
High-income North America	Prevalence	1110009.2 (1018230.6-1219394.8)	1287163.1 (1180508.3-1398684)	358.3 (328.7-393.2)	265.6 (245.6-288.3)	-1.1 (-1.25 to -0.96)
	Incidence	39935.3 (31272-51314.4)	49938.8 (36906.6-68932.5)	13.5 (10.6-17.5)	11.2 (8.7-14.8)	-0.72 (-0.82 to -0.62)
	YLDs	305170.7 (212792-396580.2)	341270.9 (239780-440625.9)	98.9 (69.1-128.9)	71.5 (49.7-92.2)	-1.16 (-1.3 to -1.02)
North Africa and Middle East	Prevalence	734570.9 (560816.9-1088070.9)	1579589.3 (1227268.8-2140602)	248.2 (197-352.5)	255.6 (199.6-344.3)	0.03 (-0.03 to 0.09)
	Incidence	31028.1 (24718.5-39925.2)	59433.7 (43804.6-80936.2)	9.3 (7.4-11.8)	9.6 (7.1-12.9)	1.57 (0.99 to 2.15)
	YLDs	261439.9 (170130.5-405528.6)	483112 (314464.2-721591.8)	87.3 (59.2-131.9)	77.5 (50.6-115.3)	-0.48 (-0.54 to -0.41)
Oceania	Prevalence	4758.6 (4383.4-5176.3)	14783.8 (13325.9-16629)	91.2 (84.5-98.6)	123.1 (111.1-137.3)	0.93 (0.81 to 1.06)
	Incidence	267.5 (214.2-342)	710.4 (563.1-932.5)	4.5 (3.6-5.8)	5.6 (4.3-7.4)	-0.08 (-1 to 0.85)
	YLDs	1778 (1287.5-2293.1)	5341.4 (3851.5-6863.4)	33.5 (24.3-42.7)	43.8 (31.7-56)	0.87 (0.75 to 1)
South Asia	Prevalence	1082987.9 (987002.2-1193696.4)	2282533.3 (2062017.3-2535920.5)	121.1 (110.8-132.3)	127.8 (115.9-141.5)	0.03 (-0.06 to 0.12)
	Incidence	66694.5 (51731.3-88547.9)	104040.6 (77913.7-142614.4)	7 (5.3-9.5)	6.1 (4.5-8.5)	-0.64 (-0.92 to -0.36)
	YLDs	406796.8 (287869.7-519753.4)	755596.3 (545580.5-980258.7)	44.7 (32-57)	41.9 (30.4-54.2)	-0.37 (-0.49 to -0.26)
Southeast Asia	Prevalence	581381.8 (486355.8-782562.7)	961197.3 (842887.5-1150626.7)	140.7 (121.8-179)	130.2 (114.6-155.2)	-0.24 (-0.31 to -0.18)
	Incidence	29485.9 (23480.1-37749.5)	34341.2 (27164.7-43807.5)	6.5 (5.1-8.3)	5 (3.9-6.4)	-0.82 (-1.59 to -0.04)
	YLDs	213812.9 (145149.3-315523.3)	310472.4 (218905.2-420978)	51 (35.2-72.9)	41.9 (29.7-56.6)	-0.63 (-0.7 to -0.56)
Southern Latin America	Prevalence	144877.4 (135438.9-157534.1)	232966.8 (215861-253505.9)	300.5 (280.6-326.1)	308.7 (285.1-337.1)	0.13 (-0.02 to 0.29)
	Incidence	5574.4 (4437.5-6942.7)	7377.5 (5796.7-9352.8)	11.3 (9-14.1)	10.6 (8.4-13.4)	-0.17 (-0.27 to -0.07)
	YLDs	48773 (35084.9-61821)	69217.2 (49337-89601.3)	101 (72.6-128.1)	92.2 (65.5-119.3)	-0.24 (-0.38 to -0.1)
Southern Sub-Saharan Africa	Prevalence	73576.4 (66999.7-83690.7)	82587.6 (76242.5-90145.5)	174.8 (159.6-193.3)	104 (96.1-113.7)	-1.81 (-2.04 to -1.57)
	Incidence	3726.5 (2857.7-4951.2)	4340.9 (3356.6-5640.9)	7.5 (5.7-9.9)	5.3 (4.1-6.8)	-1.29 (-1.4 to -1.18)
	YLDs	26271 (18679.9-34131.6)	27430.8 (19839.2-34683.9)	61.5 (43.8-79)	34.2 (24.7-43.2)	-2.04 (-2.26 to -1.82)
Tropical Latin America	Prevalence	320797.1 (288706.5-354740.1)	540300.7 (492871.3-591867.5)	239.8 (216.8-263.2)	214.1 (194.5-235.4)	-0.51 (-0.63 to -0.39)
	Incidence	14795.6 (11391.5-19351.2)	19289.6 (14864.7-25414.2)	9.9 (7.6-13)	8.2 (6.3-10.7)	-0.25 (-0.37 to -0.13)
	YLDs	111738.3 (80607.5-144028.3)	165987.1 (119029.2-214556.6)	82.5 (60-105.7)	65.9 (47.2-85.5)	-0.85 (-0.96 to -0.74)
Western Europe	Prevalence	1793952.8 (1642493.2-1974084.5)	1781647 (1619825.8-1979784.5)	403.4 (366.6-444)	313.9 (284-350.5)	-0.87 (-0.95 to -0.78)
	Incidence	60735.6 (46471.7-81911.5)	55447.1 (38794.5-82255.9)	14.8 (11.5-19.6)	10.8 (8-14.8)	-1.12 (-1.18 to -1.06)
	YLDs	507283.7 (361303.6-664165.6)	487322.3 (340387-645822.2)	115.2 (81.7-151.5)	87.6 (61.2-116.2)	-0.9 (-0.98 to -0.82)
Western Sub-Saharan Africa	Prevalence	121711.3 (111999.5-132477.1)	345792.9 (304339.3-400199.5)	81.8 (75.9-88)	91.8 (82-105)	0.37 (0.31 to 0.43)
	Incidence	8258.4 (6491.6-10572.3)	18330.8 (14634.6-23499.8)	4.5 (3.6-5.9)	4.2 (3.3-5.4)	-0.31 (-0.62 to -0.01)
	YLDs	46533.1 (32822.8-59128.1)	124720.4 (87336.9-166761.4)	30.7 (21.9-39)	32.5 (23-43)	0.17 (0.11 to 0.23)

Supplementary Table 2. APCs and AAPCs of age-standardized incidence of SCI in China during 1990-2021

Segments	Age-standardized Incidence		
	Year	APC (95% CI)	p-value
Both			
Trend 1	1990-2011	-0.98 (-1.37 - -0.59)	<0.01
Trend 2	2011-2021	2.05 (0.69 - 3.42)	<0.01
AAPC	1990-2021	-0.02 (-0.50 - 0.47)	0.95
Female			
Trend 1	1990-2011	-1.25 (-1.83 - -0.67)	<0.01
Trend 2	2011-2021	1.97 (-0.06 - 4.03)	0.06
AAPC	1990-2021	-0.22 (-0.94 - 0.50)	0.55
Male			
Trend 1	1990-2001	-0.00 (-0.57 - 0.57)	0.99
Trend 2	2001-2006	-3.33 (-5.79 - -0.81)	0.01
Trend 3	2006-2021	1.43 (1.06 - 1.81)	<0.01
AAPC	1990-2021	0.14 (-0.33 - 0.61)	0.55

APCs, annual percent changes; AAPCs, average annual percent changes.

Supplementary Table 3. APCs and AAPCs of age-standardized prevalence of SCI in China during 1990-2021

Segments	Age-standardized Prevalence		
	Year	APC (95% CI)	p-value
Both			
Trend 1	1990-2001	0.11 (-0.04 - 0.26)	0.14
Trend 2	2001-2005	-4.80 (-5.75 - -3.83)	<0.01
Trend 3	2005-2010	0.27 (-0.34 - 0.88)	0.37
Trend 4	2010-2018	1.43 (1.15 - 1.71)	<0.01
Trend 5	2018-2021	2.84 (1.63 - 4.06)	<0.01
AAPC	1990-2021	0.09 (-0.12 - 0.29)	0.41
Female			
Trend 1	1990-2001	0.04 (-0.09 - 0.16)	0.57
Trend 2	2001-2004	-6.99 (-8.54 - -5.41)	<0.01
Trend 3	2004-2007	-1.86 (-3.32 - -0.38)	0.02
Trend 4	2007-2018	1.29 (1.15 - 1.43)	<0.01
Trend 5	2018-2021	3.60 (2.43 - 4.80)	<0.01
AAPC	1990-2021	-0.07 (-0.31 - 0.17)	0.55
Male			
Trend 1	1990-1995	-0.20 (-0.67 - 0.27)	0.37
Trend 2	1995-2000	0.88 (0.21 - 1.56)	0.01
Trend 3	2000-2005	-3.32 (-3.92 - -2.72)	<0.01
Trend 4	2005-2009	-0.14 (-1.06 - 0.79)	0.75
Trend 5	2009-2018	1.36 (1.15 - 1.57)	<0.01
Trend 6	2018-2021	2.55 (1.46 - 3.66)	<0.01
AAPC	1990-2021	0.18 (-0.04 - 0.40)	0.10

APCs, annual percent changes; AAPCs, average annual percent changes.

Supplementary Table 4. APCs and AAPCs of age-standardized YLD of SCI in China during 1990-2021

Segments	Age-standardized YLD		
	Year	APC (95% CI)	p-value
Both			
Trend 1	1990-2001	-0.56 (-0.69 - -0.42)	<0.01
Trend 2	2001-2005	-5.97 (-6.89 - -5.03)	<0.01
Trend 3	2005-2010	-1.01 (-1.64 - -0.37)	<0.01
Trend 4	2010-2018	0.88 (0.61 - 1.16)	<0.01
Trend 5	2018-2021	2.49 (1.42 - 3.58)	<0.01
AAPC	1990-2021	-0.69 (-0.88 - -0.49)	<0.01
Female			
Trend 1	1990-2000	-0.42 (-0.58 - -0.27)	<0.01
Trend 2	2000-2005	-6.27 (-6.87 - -5.67)	<0.01
Trend 3	2005-2008	-2.04 (-4.00 - -0.03)	0.05
Trend 4	2008-2018	0.66 (0.47 - 0.86)	<0.01
Trend 5	2018-2021	3.33 (2.20 - 4.48)	<0.01
AAPC	1990-2021	-0.85 (-1.08 - -0.61)	<0.01
Male			
Trend 1	1990-2001	-0.44 (-0.59 - -0.29)	<0.01
Trend 2	2001-2005	-5.01 (-6.05 - -3.96)	<0.01
Trend 3	2005-2010	-1.06 (-1.76 - -0.36)	0.01
Trend 4	2010-2018	0.90 (0.59 - 1.20)	<0.01
Trend 5	2018-2021	2.11 (0.91 - 3.32)	<0.01
AAPC	1990-2021	-0.56 (-0.78 - -0.34)	<0.01

APCs, annual percent changes; AAPCs, average annual percent changes.

Supplementary Table 5. APCs and AAPCs of age-standardized incidence of SCI in global during 1990-2021

Segments	Age-standardized Incidence		
	Year	APC (95% CI)	p-value
Both			
Trend 1	1990-2021	-0.81 (-0.94 - -0.69)	<0.01
AAPC	1990-2021	-0.81 (-0.94 - -0.69)	<0.01
Female			
Trend 1	1990-2021	-0.79 (-1.03 - -0.54)	<0.01
AAPC	1990-2021	-0.79 (-1.03 - -0.54)	<0.01
Male			
Trend 1	1990-2021	-0.82 (-0.91 - -0.72)	<0.01
AAPC	1990-2021	-0.82 (-0.91 - -0.72)	<0.01

APCs, annual percent changes; AAPCs, average annual percent changes.

Supplementary Table 6. APCs and AAPCs of age-standardized prevalence of SCI in global during 1990-2021

Segments	Age-standardized Prevalence		
	Year	APC (95% CI)	p-value
Both			
Trend 1	1990-2000	-0.37 (-0.41 - -0.34)	<0.01
Trend 2	2000-2005	-1.34 (-1.51 - -1.17)	<0.01
Trend 3	2005-2010	-0.40 (-0.56 - -0.23)	<0.01
Trend 4	2010-2015	-1.16 (-1.33 - -0.99)	<0.01
Trend 5	2015-2018	-0.51 (-1.08 - 0.06)	0.08
Trend 6	2018-2021	0.33 (0.03 - 0.62)	0.03
AAPC	1990-2021	-0.61 (-0.68 - -0.53)	<0.01
Female			
Trend 1	1990-2000	-0.25 (-0.29 - -0.21)	<0.01
Trend 2	2000-2004	-1.56 (-1.83 - -1.29)	<0.01
Trend 3	2004-2011	-0.33 (-0.43 - -0.24)	<0.01
Trend 4	2011-2014	-1.41 (-1.97 - -0.85)	<0.01
Trend 5	2014-2018	-0.63 (-0.92 - -0.34)	<0.01
Trend 6	2018-2021	0.45 (0.14 - 0.76)	0.01
AAPC	1990-2021	-0.53 (-0.61 - -0.45)	<0.01
Male			
Trend 1	1990-1994	-0.67 (-0.84 - -0.50)	<0.01
Trend 2	1994-2000	-0.28 (-0.41 - -0.16)	<0.01
Trend 3	2000-2005	-1.33 (-1.52 - -1.15)	<0.01
Trend 4	2005-2010	-0.52 (-0.70 - -0.33)	<0.01
Trend 5	2010-2016	-1.12 (-1.25 - -0.99)	<0.01
Trend 6	2016-2021	0.01 (-0.13 - 0.15)	0.85
AAPC	1990-2021	-0.66 (-0.71 - -0.60)	<0.01

APCs, annual percent changes; AAPCs, average annual percent changes.

Supplementary Table 7. APCs and AAPCs of age-standardized YLD of SCI in global during 1990-2021

Segments	Age-standardized YLD		
	Year	APC (95% CI)	p-value
Both			
Trend 1	1990-2000	-0.57 (-0.61 - -0.53)	<0.01
Trend 2	2000-2005	-1.61 (-1.78 - -1.43)	<0.01
Trend 3	2005-2011	-0.80 (-0.93 - -0.68)	<0.01
Trend 4	2011-2014	-1.64 (-2.20 - -1.07)	<0.01
Trend 5	2014-2018	-0.87 (-1.15 - -0.58)	<0.01
Trend 6	2018-2021	0.08 (-0.20 - 0.37)	0.54
AAPC	1990-2021	-0.86 (-0.94 - -0.79)	<0.01
Female			
Trend 1	1990-2000	-0.44 (-0.48 - -0.40)	<0.01
Trend 2	2000-2004	-1.87 (-2.11 - -1.62)	<0.01
Trend 3	2004-2011	-0.66 (-0.74 - -0.58)	<0.01
Trend 4	2011-2014	-1.65 (-2.13 - -1.16)	<0.01
Trend 5	2014-2018	-0.91 (-1.15 - -0.66)	<0.01
Trend 6	2018-2021	0.12 (-0.13 - 0.37)	0.32
AAPC	1990-2021	-0.80 (-0.86 - -0.73)	<0.01
Male			
Trend 1	1990-1994	-0.92 (-1.09 - -0.75)	<0.01
Trend 2	1994-2000	-0.46 (-0.58 - -0.33)	<0.01
Trend 3	2000-2005	-1.61 (-1.78 - -1.44)	<0.01
Trend 4	2005-2010	-0.90 (-1.08 - -0.72)	<0.01
Trend 5	2010-2017	-1.27 (-1.36 - -1.17)	<0.01
Trend 6	2017-2021	-0.03 (-0.20 - 0.15)	0.76
AAPC	1990-2021	-0.90 (-0.96 - -0.85)	<0.01

APCs, annual percent changes; AAPCs, average annual percent changes.

Supplementary Table 8. Age, period and cohort effects of SCI incidence in China

Factors	Effect coefficient	Standard error	Z value	P-value	95% CI	
					Lower	Upper
Age						
< 5	0.05	0.02	2.65	0.01	0.01	0.08
5-9	-0.47	0.02	-29.67	<0.01	-0.50	-0.44
10-14	-0.55	0.01	-38.96	<0.01	-0.58	-0.52
15-19	-0.33	0.01	-27.01	<0.01	-0.35	-0.30
20-24	-0.16	0.01	-15.13	<0.01	-0.18	-0.14
25-29	-0.19	0.01	-21.45	<0.01	-0.20	-0.17
30-34	-0.22	0.01	-30.88	<0.01	-0.24	-0.21
35-39	-0.24	0.01	-39.97	<0.01	-0.25	-0.23
40-44	-0.30	0.01	-57.12	<0.01	-0.31	-0.29
45-49	-0.36	0.00	-73.75	<0.01	-0.37	-0.35
50-54	-0.38	0.01	-72.49	<0.01	-0.39	-0.37
55-59	-0.34	0.01	-55.51	<0.01	-0.35	-0.33
60-64	-0.27	0.01	-36.71	<0.01	-0.28	-0.25
65-69	-0.19	0.01	-22.47	<0.01	-0.21	-0.18
70-74	-0.07	0.01	-6.61	<0.01	-0.09	-0.05
75-79	0.22	0.01	18.57	<0.01	0.20	0.24
80-84	0.63	0.01	46.20	<0.01	0.60	0.65
85-89	0.94	0.02	58.44	<0.01	0.90	0.97
90-94	1.08	0.02	51.80	<0.01	1.04	1.12
95+	1.15	0.04	30.88	<0.01	1.08	1.22
Period						
1992-1996	-0.08	0.01	-15.08	<0.01	-0.09	-0.07
1997-2001	-0.03	0.00	-8.62	<0.01	-0.04	-0.02
2002-2006	-0.10	0.00	-40.07	<0.01	-0.11	-0.10
2007-2011	-0.04	0.00	-16.54	<0.01	-0.05	-0.04
2012-2016	0.05	0.00	13.49	<0.01	0.04	0.05
2017-2021	0.21	0.00	41.78	<0.01	0.20	0.21
Cohort						
1897-1901	-0.16	0.22	-0.73	0.47	-0.60	0.27
1902-1906	-0.03	0.07	-0.42	0.67	-0.18	0.11
1907-1911	0.04	0.04	1.02	0.31	-0.04	0.12
1912-1916	0.12	0.03	3.78	<0.01	0.06	0.18
1917-1921	0.20	0.03	7.24	<0.01	0.14	0.25
1922-1926	0.27	0.02	11.10	<0.01	0.22	0.32
1927-1931	0.33	0.02	14.59	<0.01	0.28	0.37
1932-1936	0.37	0.02	18.20	<0.01	0.33	0.41
1937-1941	0.38	0.02	20.29	<0.01	0.34	0.42
1942-1946	0.37	0.02	21.62	<0.01	0.33	0.40
1947-1951	0.36	0.02	23.92	<0.01	0.34	0.39
1952-1956	0.35	0.01	25.86	<0.01	0.32	0.37
1957-1961	0.32	0.01	27.06	<0.01	0.30	0.34
1962-1966	0.27	0.01	26.69	<0.01	0.25	0.29
1967-1971	0.21	0.01	24.69	<0.01	0.19	0.22
1972-1976	0.13	0.01	19.30	<0.01	0.12	0.15
1977-1981	0.06	0.01	10.22	<0.01	0.05	0.07
1982-1986	-0.02	0.00	-3.99	<0.01	-0.03	-0.01
1987-1991	-0.13	0.00	-28.67	<0.01	-0.13	-0.12
1992-1996	-0.20	0.00	-42.40	<0.01	-0.21	-0.19
1997-2001	-0.31	0.01	-46.91	<0.01	-0.32	-0.30
2002-2006	-0.46	0.01	-51.94	<0.01	-0.48	-0.44
2007-2011	-0.55	0.01	-51.64	<0.01	-0.58	-0.53
2012-2016	-0.82	0.01	-61.40	<0.01	-0.85	-0.80
2017-2021	-1.09	0.02	-60.27	<0.01	-1.12	-1.05
Intercept	-9.60	0.01	-901.64	<0.01	-9.62	-9.58
Log likelihood	-23218.54					
AIC	129.26					
BIC	41300.42					

Supplementary Table 9. Age, period and cohort effects of SCI prevalence in China

Factors	Effect coefficient	Standard error	Z value	P-value	95% CI	
					Lower	Upper
Age						
< 5	-1.60	0.01	-168.97	<0.01	-1.62	-1.58
5-9	-0.91	0.01	-109.74	<0.01	-0.92	-0.89
10-14	-0.60	0.01	-82.78	<0.01	-0.62	-0.59
15-19	-0.36	0.01	-57.05	<0.01	-0.37	-0.35
20-24	-0.11	0.01	-20.60	<0.01	-0.12	-0.10
25-29	0.08	0.00	18.49	<0.01	0.07	0.09
30-34	0.21	0.00	61.90	<0.01	0.21	0.22
35-39	0.31	0.00	119.38	<0.01	0.30	0.31
40-44	0.37	0.00	208.90	<0.01	0.37	0.38
45-49	0.42	0.00	321.36	<0.01	0.41	0.42
50-54	0.43	0.00	301.80	<0.01	0.43	0.43
55-59	0.42	0.00	203.99	<0.01	0.42	0.43
60-64	0.42	0.00	143.50	<0.01	0.41	0.42
65-69	0.44	0.00	115.47	<0.01	0.43	0.45
70-74	0.42	0.00	87.81	<0.01	0.41	0.43
75-79	0.35	0.01	60.81	<0.01	0.34	0.36
80-84	0.22	0.01	32.09	<0.01	0.20	0.23
85-89	-0.02	0.01	-2.21	0.03	-0.03	0.00
90-94	-0.19	0.01	-18.26	<0.01	-0.21	-0.17
95+	-0.30	0.02	-16.29	<0.01	-0.34	-0.26
Period						
1992-1996	-0.06	0.00	-24.83	<0.01	-0.07	-0.06
1997-2001	-0.01	0.00	-5.41	<0.01	-0.01	-0.01
2002-2006	-0.10	0.00	-144.15	<0.01	-0.10	-0.10
2007-2011	-0.08	0.00	-126.95	<0.01	-0.09	-0.08
2012-2016	0.05	0.00	30.41	<0.01	0.04	0.05
2017-2021	0.21	0.00	83.74	<0.01	0.20	0.21
Cohort						
1897-1901	-0.39	0.12	-3.15	<0.01	-0.63	-0.15
1902-1906	-0.23	0.04	-6.02	<0.01	-0.30	-0.15
1907-1911	-0.04	0.02	-2.27	0.02	-0.08	-0.01
1912-1916	0.14	0.02	8.94	<0.01	0.11	0.17
1917-1921	0.28	0.01	19.88	<0.01	0.25	0.30
1922-1926	0.38	0.01	29.73	<0.01	0.35	0.40
1927-1931	0.45	0.01	38.23	<0.01	0.43	0.47
1932-1936	0.49	0.01	45.76	<0.01	0.47	0.51
1937-1941	0.49	0.01	50.57	<0.01	0.48	0.51
1942-1946	0.48	0.01	54.44	<0.01	0.46	0.50
1947-1951	0.47	0.01	59.23	<0.01	0.45	0.48
1952-1956	0.42	0.01	61.52	<0.01	0.41	0.44
1957-1961	0.38	0.01	63.39	<0.01	0.37	0.39
1962-1966	0.31	0.00	61.34	<0.01	0.30	0.32
1967-1971	0.22	0.00	55.52	<0.01	0.22	0.23
1972-1976	0.14	0.00	45.32	<0.01	0.14	0.15
1977-1981	0.05	0.00	22.90	<0.01	0.05	0.06
1982-1986	-0.04	0.00	-24.04	<0.01	-0.04	-0.03
1987-1991	-0.15	0.00	-130.88	<0.01	-0.16	-0.15
1992-1996	-0.26	0.00	-164.97	<0.01	-0.27	-0.26
1997-2001	-0.39	0.00	-151.62	<0.01	-0.39	-0.38
2002-2006	-0.55	0.00	-151.63	<0.01	-0.56	-0.54
2007-2011	-0.63	0.00	-135.13	<0.01	-0.64	-0.62
2012-2016	-0.91	0.01	-146.74	<0.01	-0.93	-0.90
2017-2021	-1.11	0.01	-112.28	<0.01	-1.13	-1.09
Intercept	-6.76	0.01	-1164.49	<0.01	-6.77	-6.75
Log likelihood	-456759.01					
AIC	2537.82					
BIC	907353.30					

Supplementary Table 10. Age, period and cohort effects of SCI incidence in Global

Factors	Effect coefficient	Standard error	Z value	P-value	95% CI	
					Lower	Upper
Age						
< 5	-0.41	0.00	-113.42	<0.01	-0.41	-0.40
5-9	-0.46	0.00	-144.12	<0.01	-0.47	-0.46
10-14	-0.49	0.00	-167.43	<0.01	-0.49	-0.48
15-19	0.07	0.00	30.29	<0.01	0.07	0.08
20-24	0.11	0.00	49.56	<0.01	0.10	0.11
25-29	-0.07	0.00	-33.76	<0.01	-0.07	-0.06
30-34	-0.18	0.00	-92.03	<0.01	-0.18	-0.17
35-39	-0.27	0.00	-140.26	<0.01	-0.27	-0.26
40-44	-0.35	0.00	-180.97	<0.01	-0.35	-0.35
45-49	-0.38	0.00	-189.08	<0.01	-0.39	-0.38
50-54	-0.35	0.00	-163.80	<0.01	-0.36	-0.35
55-59	-0.30	0.00	-132.49	<0.01	-0.31	-0.30
60-64	-0.24	0.00	-96.87	<0.01	-0.24	-0.23
65-69	-0.12	0.00	-46.15	<0.01	-0.13	-0.12
70-74	0.05	0.00	18.60	<0.01	0.05	0.06
75-79	0.30	0.00	95.78	<0.01	0.29	0.31
80-84	0.58	0.00	165.50	<0.01	0.57	0.58
85-89	0.78	0.00	189.19	<0.01	0.78	0.79
90-94	0.87	0.01	153.39	<0.01	0.86	0.88
95+	0.85	0.01	85.66	<0.01	0.83	0.87
Period						
1992-1996	0.01	0.00	12.04	<0.01	0.01	0.02
1997-2001	0.00	0.00	3.65	<0.01	0.00	0.01
2002-2006	-0.02	0.00	-26.66	<0.01	-0.03	-0.02
2007-2011	-0.01	0.00	-14.28	<0.01	-0.01	-0.01
2012-2016	0.00	0.00	3.47	<0.01	0.00	0.01
2017-2021	0.02	0.00	12.83	<0.01	0.01	0.02
Cohort						
1897-1901	-0.02	0.04	-0.49	0.62	-0.10	0.06
1902-1906	0.08	0.02	4.66	<0.01	0.04	0.11
1907-1911	0.15	0.01	15.51	<0.01	0.13	0.16
1912-1916	0.20	0.01	28.09	<0.01	0.18	0.21
1917-1921	0.21	0.01	35.05	<0.01	0.20	0.22
1922-1926	0.25	0.01	48.03	<0.01	0.24	0.26
1927-1931	0.25	0.00	52.48	<0.01	0.24	0.26
1932-1936	0.26	0.00	58.56	<0.01	0.25	0.26
1937-1941	0.26	0.00	63.60	<0.01	0.25	0.27
1942-1946	0.25	0.00	66.32	<0.01	0.24	0.26
1947-1951	0.24	0.00	68.74	<0.01	0.23	0.25
1952-1956	0.22	0.00	70.42	<0.01	0.22	0.23
1957-1961	0.19	0.00	67.38	<0.01	0.19	0.20
1962-1966	0.13	0.00	52.17	<0.01	0.13	0.14
1967-1971	0.05	0.00	23.36	<0.01	0.05	0.06
1972-1976	0.00	0.00	-0.36	0.72	0.00	0.00
1977-1981	-0.05	0.00	-27.12	<0.01	-0.05	-0.04
1982-1986	-0.10	0.00	-63.27	<0.01	-0.10	-0.10
1987-1991	-0.19	0.00	-126.24	<0.01	-0.20	-0.19
1992-1996	-0.22	0.00	-141.46	<0.01	-0.22	-0.21
1997-2001	-0.25	0.00	-135.82	<0.01	-0.25	-0.24
2002-2006	-0.32	0.00	-142.83	<0.01	-0.32	-0.31
2007-2011	-0.38	0.00	-133.34	<0.01	-0.38	-0.37
2012-2016	-0.52	0.00	-147.22	<0.01	-0.53	-0.52
2017-2021	-0.69	0.01	-128.07	<0.01	-0.70	-0.68
Intercept	-9.23	0.00	-4717.23	<0.01	-9.23	-9.22
Log likelihood	-215803.01					
AIC	1199.17					
BIC	425740.70					

Supplementary Table 11. Age, period and cohort effects of SCI prevalence in Global

Factors	Effect coefficient	Standard error	Z value	P-value	95% CI	
					Lower	Upper
Age						
< 5	-2.16	0.00	-1311.91	<0.01	-2.16	-2.15
5-9	-1.21	0.00	-939.60	<0.01	-1.21	-1.21
10-14	-0.73	0.00	-658.78	<0.01	-0.73	-0.73
15-19	-0.35	0.00	-363.68	<0.01	-0.35	-0.35
20-24	0.00	0.00	-0.67	0.50	0.00	0.00
25-29	0.21	0.00	302.50	<0.01	0.21	0.21
30-34	0.33	0.00	576.98	<0.01	0.33	0.33
35-39	0.41	0.00	846.72	<0.01	0.40	0.41
40-44	0.45	0.00	1076.31	<0.01	0.45	0.45
45-49	0.47	0.00	1153.72	<0.01	0.47	0.47
50-54	0.47	0.00	1056.17	<0.01	0.47	0.47
55-59	0.44	0.00	849.09	<0.01	0.44	0.44
60-64	0.42	0.00	676.94	<0.01	0.42	0.42
65-69	0.44	0.00	599.39	<0.01	0.44	0.44
70-74	0.43	0.00	501.35	<0.01	0.43	0.43
75-79	0.40	0.00	394.63	<0.01	0.40	0.40
80-84	0.33	0.00	276.00	<0.01	0.32	0.33
85-89	0.15	0.00	105.06	<0.01	0.15	0.16
90-94	-0.08	0.00	-39.44	<0.01	-0.09	-0.08
95+	-0.41	0.00	-99.42	<0.01	-0.42	-0.40
Period						
1992-1996	-0.04	0.00	-90.40	<0.01	-0.04	-0.03
1997-2001	-0.02	0.00	-58.23	<0.01	-0.02	-0.02
2002-2006	-0.02	0.00	-110.24	<0.01	-0.02	-0.02
2007-2011	0.00	0.00	4.22	<0.01	0.00	0.00
2012-2016	0.01	0.00	51.79	<0.01	0.01	0.01
2017-2021	0.06	0.00	150.17	<0.01	0.06	0.06
Cohort						
1897-1901	0.04	0.02	2.09	0.04	0.00	0.07
1902-1906	0.13	0.01	20.47	<0.01	0.12	0.14
1907-1911	0.22	0.00	63.18	<0.01	0.21	0.22
1912-1916	0.30	0.00	118.21	<0.01	0.29	0.30
1917-1921	0.32	0.00	148.68	<0.01	0.32	0.33
1922-1926	0.37	0.00	191.43	<0.01	0.37	0.37
1927-1931	0.38	0.00	215.60	<0.01	0.38	0.38
1932-1936	0.37	0.00	228.15	<0.01	0.37	0.37
1937-1941	0.35	0.00	237.39	<0.01	0.35	0.35
1942-1946	0.32	0.00	241.38	<0.01	0.32	0.33
1947-1951	0.29	0.00	242.57	<0.01	0.29	0.29
1952-1956	0.24	0.00	222.43	<0.01	0.23	0.24
1957-1961	0.20	0.00	210.35	<0.01	0.19	0.20
1962-1966	0.16	0.00	205.81	<0.01	0.16	0.17
1967-1971	0.10	0.00	146.40	<0.01	0.10	0.10
1972-1976	0.01	0.00	12.85	<0.01	0.01	0.01
1977-1981	-0.07	0.00	-135.65	<0.01	-0.07	-0.06
1982-1986	-0.14	0.00	-330.33	<0.01	-0.14	-0.14
1987-1991	-0.25	0.00	-587.95	<0.01	-0.25	-0.25
1992-1996	-0.32	0.00	-667.00	<0.01	-0.32	-0.32
1997-2001	-0.40	0.00	-655.98	<0.01	-0.40	-0.40
2002-2006	-0.49	0.00	-612.71	<0.01	-0.49	-0.49
2007-2011	-0.56	0.00	-537.95	<0.01	-0.56	-0.56
2012-2016	-0.71	0.00	-472.49	<0.01	-0.71	-0.71
2017-2021	-0.85	0.00	-284.70	<0.01	-0.86	-0.85
Intercept	-6.32	0.00	-7673.41	<0.01	-6.32	-6.31
Log likelihood	-3937226.68					
AIC	21873.75					
BIC	7867532.00					

Supplementary Table 12. The predictive capacity of ASIR and ASPR of SCI in china on ARIMA models.

ASIR

	ME	RMSE	MAE	MPE	MAPE	MASE
Male	-0.009	0.237	0.153	-0.254	2.207	0.977
Female	0.000	0.495	0.382	-1.286	9.164	1.524
Both	-0.008	0.377	0.251	-0.583	4.500	1.260

ASPR

	ME	RMSE	MAE	MPE	MAPE	MASE
Male	-0.074	2.255	1.368	-0.063	0.770	0.604
Female	0.018	1.849	1.061	0.070	1.018	0.606
Both	-0.002	2.225	1.228	0.032	0.869	0.617

ME: Mean Error, RMSE: Root Mean Squared Error, MAE: Mean Absolute Error, MPE: Mean Percentage Error, MAPE: Mean Absolute Percentage Error, MASE: Mean Absolute Scaled Error

Supplementary Table 13. The predictive capacity of ASIR and ASPR of SCI in global on ARIMA models.

ASIR

	ME	RMSE	MAE	MPE	MAPE	MASE
Male	-0.005	0.255	0.206	-0.069	1.936	0.867
Female	0.000	0.319	0.252	-0.228	4.353	0.667
Both	-0.011	0.258	0.218	-0.171	2.667	0.718

ASPR

	ME	RMSE	MAE	MPE	MAPE	MASE
Male	0.014	0.794	0.501	0.006	0.199	0.268
Female	0.002	0.489	0.343	0.001	0.243	0.402
Both	0.009	0.623	0.399	0.004	0.203	0.289

ME: Mean Error, RMSE: Root Mean Squared Error, MAE: Mean Absolute Error, MPE: Mean Percentage Error, MAPE: Mean Absolute Percentage Error, MASE: Mean Absolute Scaled Error

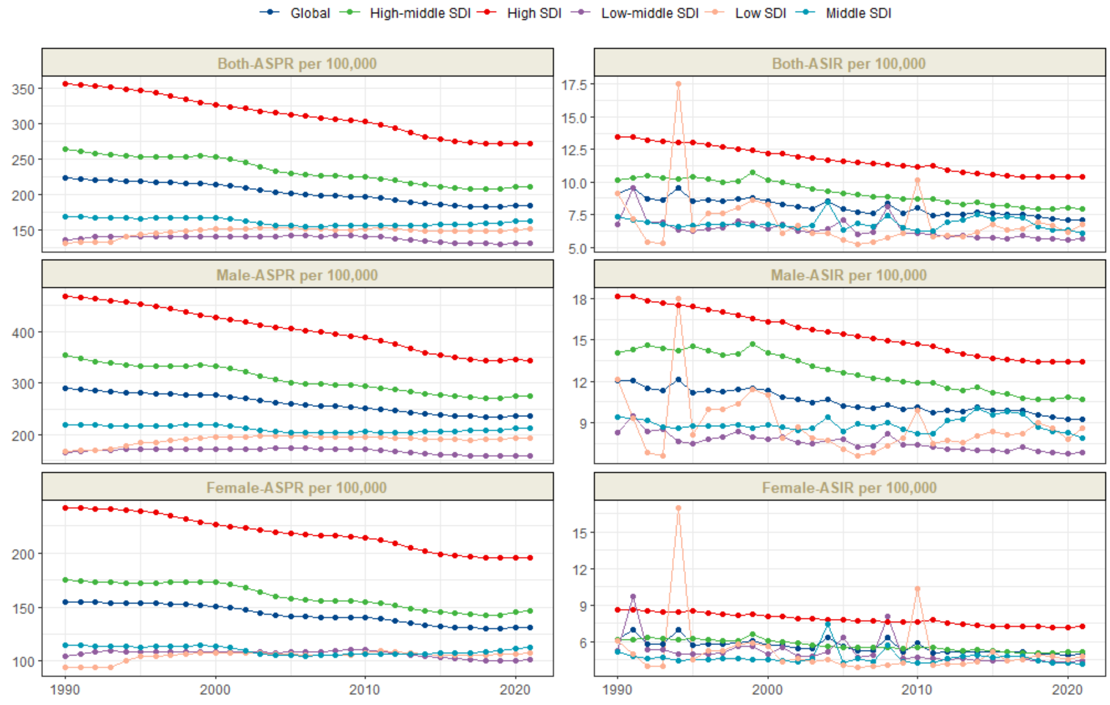

Supplementary Figure 1. Trend comparison of ASPR and ASIR of SCI in global and 5 different SDI regions from 1990 to 2021.

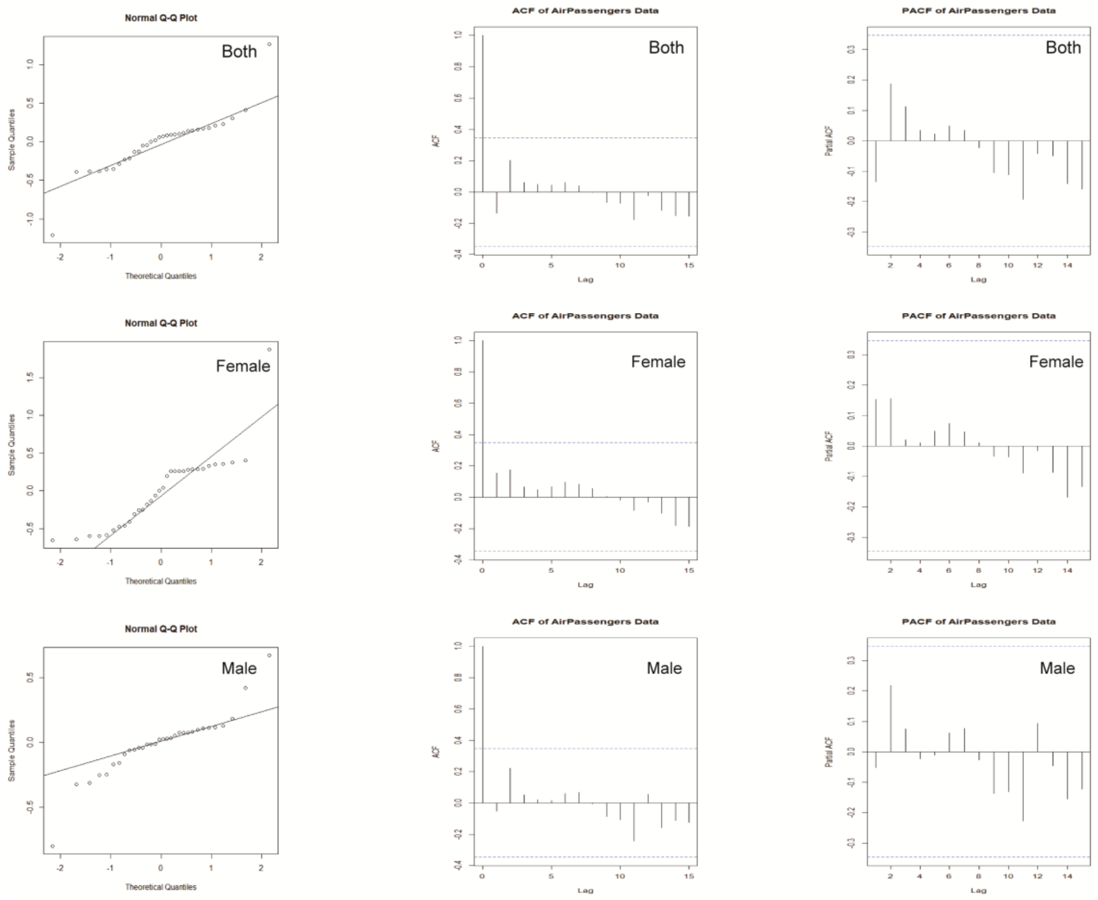

Supplementary Figure 2. Residual Q-Q plots, autocorrelation function and partial autocorrelation graphs of ARIMA models of SCI ASIR in china.

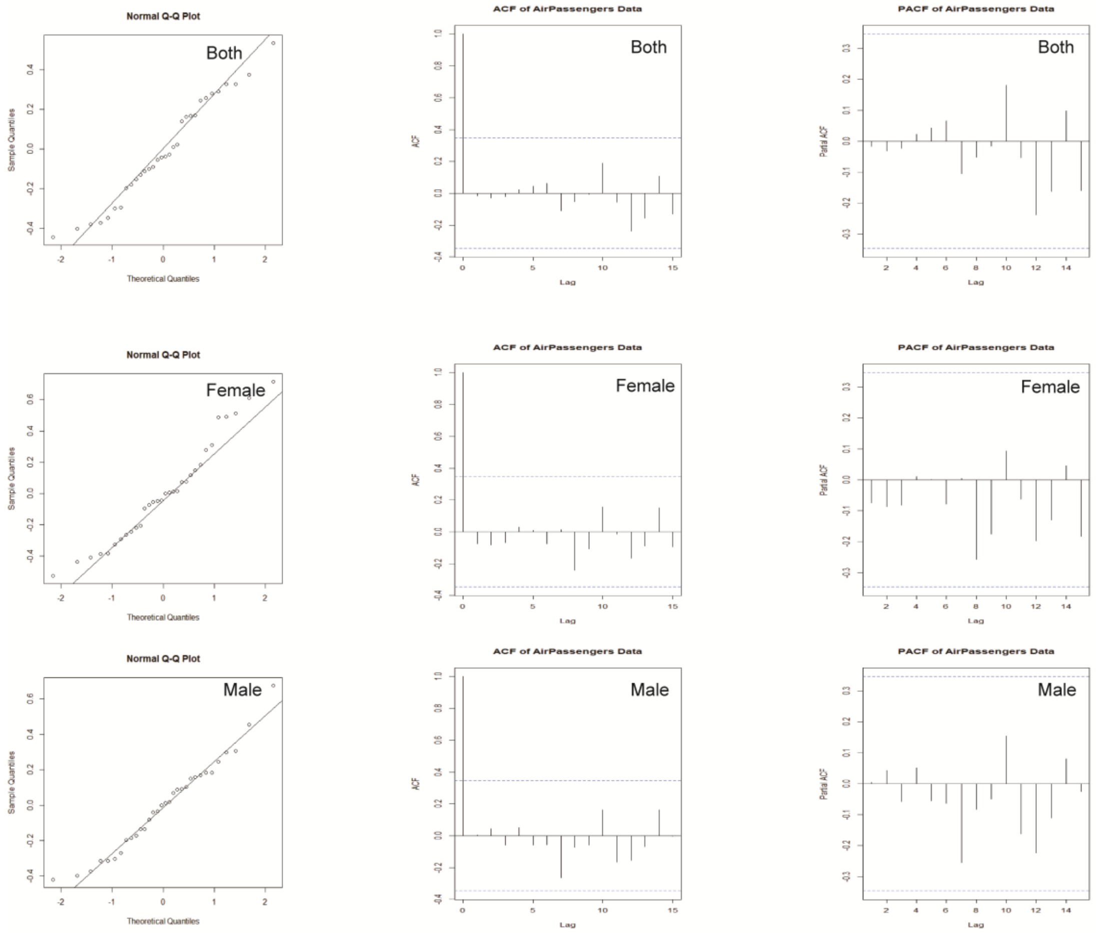

Supplementary Figure 3. Residual Q-Q plots, autocorrelation function and partial autocorrelation graphs of ARIMA models of SCI ASIR in global.

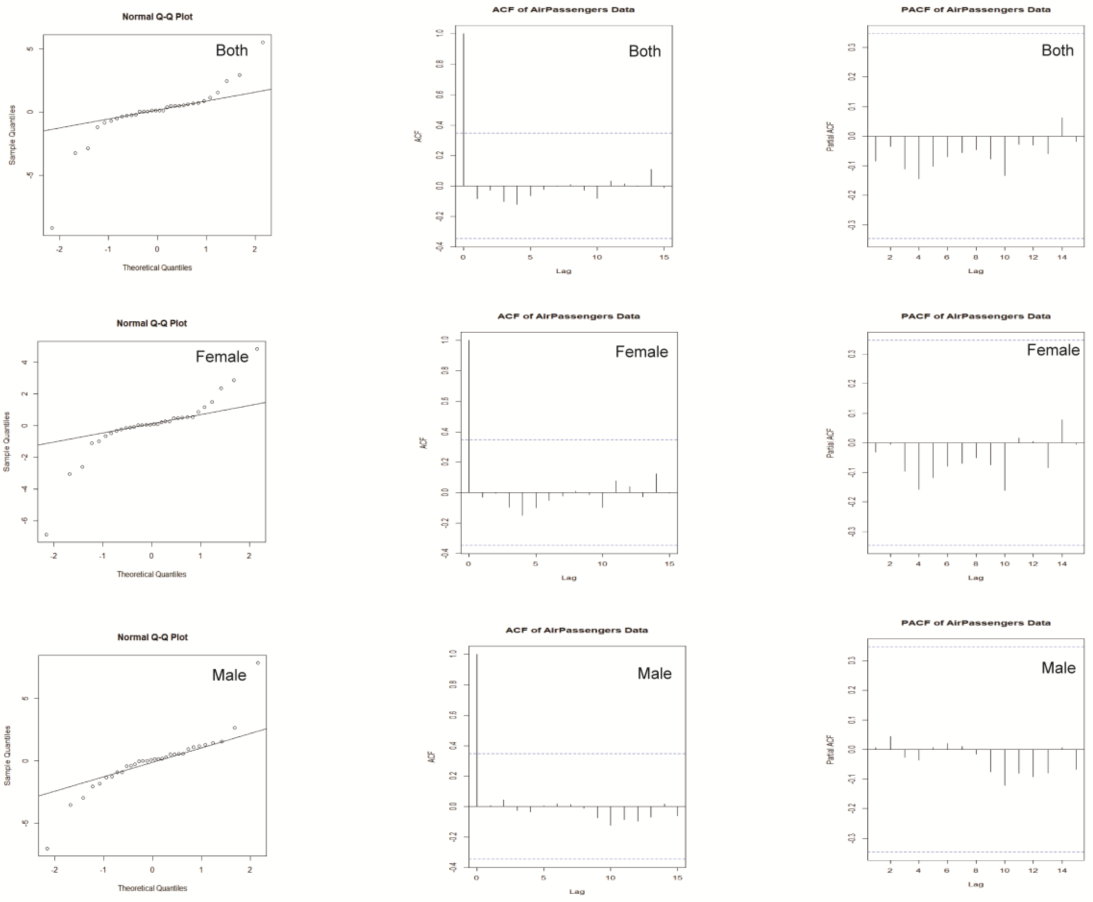

Supplementary Figure 4. Residual Q-Q plots, autocorrelation function and partial autocorrelation graphs of ARIMA models of SCI ASPR in china.

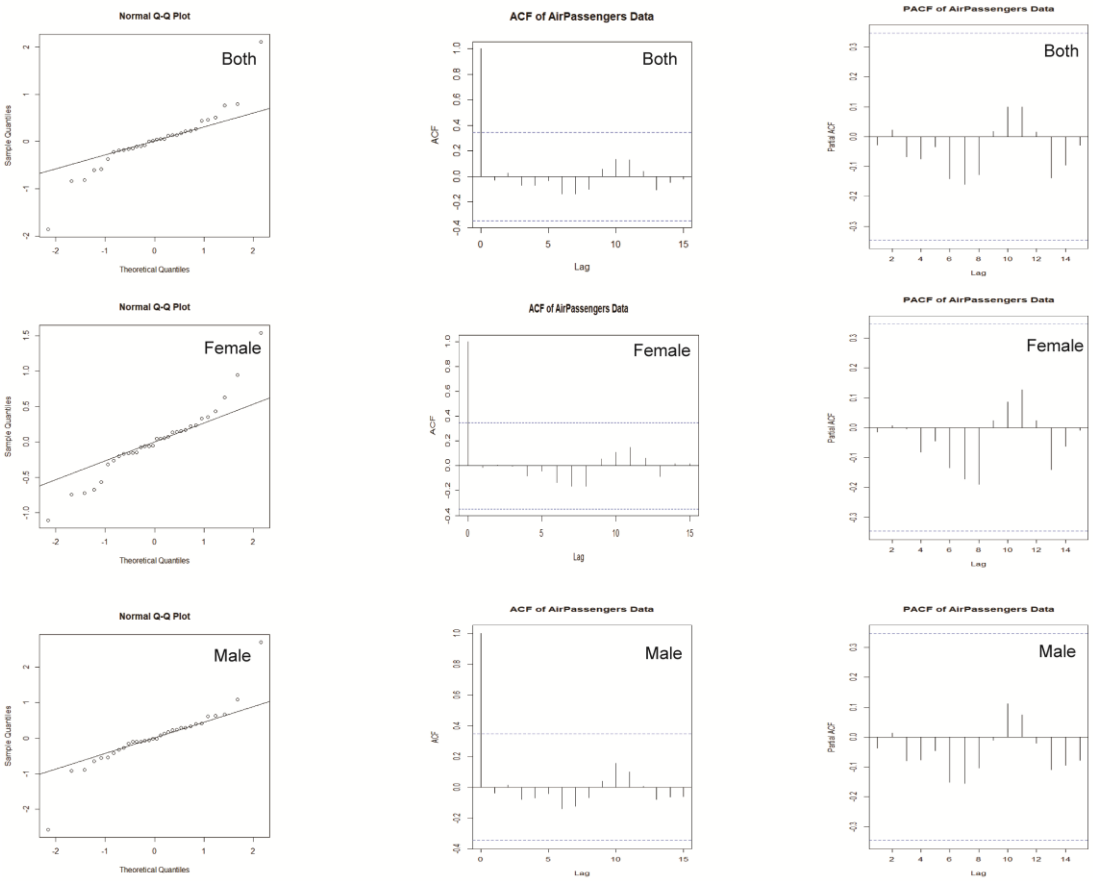

Supplementary Figure 5. Residual Q-Q plots, autocorrelation function and partial autocorrelation graphs of ARIMA models of SCI ASPR in global.

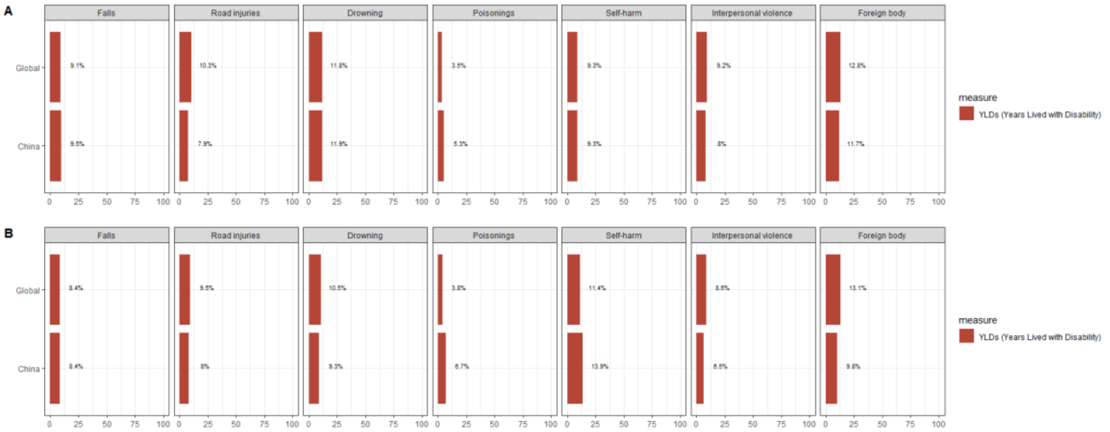

Supplementary Figure 6. Analysis of the causes of the global and China spinal cord injury burden in 1990 and 2021. (A: Analysis of the causes of the global and China spinal cord injury burden in 1990; B: Analysis of the causes of the global and China spinal cord injury burden in 2021)
