# Supplementary material for: Analysis and comparison of the trends in burden of spinal cord injury in China and worldwide from 1990 to 2021: an analysis of the global burden of disease study 2021
Source: Front Public Health. 2025 Jan 7;12:1517871. doi: 10.3389/fpubh.2024.1517871 (PMC11747465; doi:10.3389/fpubh.2024.1517871)
Supplement: Supplementary file 2 [file Data_Sheet_2.pdf]

Supplementary Table 1 ---

```
library(dplyr)
library(purrr)
dir <- "D:/XX/XX/"
files <- list.files(dir,
                    pattern = ".csv",
                    full.names = T)
data <- map_dfr(files,read.csv)
IS <- data %>%

dplyr::select(measure_name,location_name,metric_name,sex_name,age_name,rei_name,year,
cause_name,val,lower,upper) %>% ###提取需要的变量
  rename(measure=measure_name,
         location=location_name,
         metric=metric_name,
         sex=sex_name,
         age=age_name,
         cause=cause_name,
         rei=rei_name)

###YLDs/Prevalence/Incidence
###Number
###1990
IS_1990 <- IS %>%

  filter(year==1990 &
           age =='All ages' &
           metric == 'Number' &
           measure =='YLDs (Years Lived with Disability)' &
           sex =="Both" &
           rei=="Spinal Injuries"
  ) %>%

  filter(location == 'Global' |
           location == 'Low SDI' |
           location == 'Low-middle SDI' |
           location == 'Middle SDI' |
           location == 'High-middle SDI' |
           location == 'High SDI' |
           location == 'Andean Latin America' |
           location == 'Australasia' |
```

```
                  location == 'Caribbean' |
                  location == 'Central Asia' |
                  location == 'Central Europe' |
                  location == 'Central Latin America' |
                  location == 'Central Sub-Saharan Africa' |
                  location == 'East Asia' |
                  location == 'Eastern Europe' |
                  location == 'Eastern Sub-Saharan Africa' |
                  location == 'High-income Asia Pacific' |
                  location == 'High-income North America' |
                  location == 'North Africa and Middle East' |
                  location == 'Oceania' |
                  location == 'South Asia' |
                  location == 'Southeast Asia' |
                  location == 'Southern Latin America' |
                  location == 'Southern Sub-Saharan Africa' |
                  location == 'Tropical Latin America' |
                  location == 'Western Europe' |
                  location == 'Western Sub-Saharan Africa') %>%
  dplyr::select(,c(2,9:11))


IS_1990$val <- round(IS_1990$val,1)
IS_1990$lower <- round(IS_1990$lower,1)
IS_1990$upper <- round(IS_1990$upper,1)

IS_1990$'Number   1990' <- paste(IS_1990$lower,IS_1990$upper,sep = '-')
IS_1990$'Number   1990' <- paste(IS_1990$'Number   1990',')',sep = '')
IS_1990$'Number   1990' <- paste('(',IS_1990$'Number   1990',sep = '')
IS_1990$'Number   1990' <- paste(IS_1990$val,IS_1990$'Number   1990',sep = ' ')
YLDs_Num_1990 <- IS_1990[,c(1,5)]
head(YLDs_Num_1990)

## 2021
IS_2021 <- IS %>%
  filter(year ==2021 &
                  age =='All ages' &
                  metric == 'Number' &
                  measure   =='YLDs (Years Lived with Disability)' &
                  sex =="Both" &
                  rei=="Spinal Injuries"
  ) %>%
  filter(location == 'Andean Latin America' |
                  location == 'Australasia' |
```

```
                  location == 'Caribbean' |
                  location == 'Central Asia' |
                  location == 'Central Europe' |
                  location == 'Central Latin America' |
                  location == 'Central Sub-Saharan Africa' |
                  location == 'East Asia' |
                  location == 'Eastern Europe' |
                  location == 'Eastern Sub-Saharan Africa' |
                  location == 'High-income Asia Pacific' |
                  location == 'High-income North America' |
                  location == 'North Africa and Middle East' |
                  location == 'Oceania' |
                  location == 'South Asia' |
                  location == 'Southeast Asia' |
                  location == 'Southern Latin America' |
                  location == 'Southern Sub-Saharan Africa' |
                  location == 'Tropical Latin America' |
                  location == 'Western Europe' |
                  location == 'Western Sub-Saharan Africa') %>%
    dplyr::select(,c(2,9:11))


IS_2021$val <- round(IS_2021$val,1)
IS_2021$lower <- round(IS_2021$lower,1)
IS_2021$upper <- round(IS_2021$upper,1)

IS_2021$'Number    2021' <- paste(IS_2021$lower,IS_2021$upper,sep = '-')
IS_2021$'Number    2021' <- paste(IS_2021$'Number    2021',')',sep = '')
IS_2021$'Number    2021' <- paste('(',IS_2021$'Number    2021',sep = '')
IS_2021$'Number    2021' <- paste(IS_2021$val,IS_2021$'Number    2021',sep = ' ')
YLDs_Num_2021 <- IS_2021[,c(1,5)]
head(YLDs_Num_2021)



##### Age-standardized rates
## 1990
IS_1990 <- IS %>%

    filter(year==1990 &
                  age =='Age-standardized' &
                  metric == 'Rate' &
                  measure =='YLDs (Years Lived with Disability)' &
                  sex =="Both" &
```

```
                rei=="Spinal Injuries"
   ) %>%

   filter(location == 'Andean Latin America' |
                location == 'Australasia' |
                location == 'Caribbean' |
                location == 'Central Asia' |
                location == 'Central Europe' |
                location == 'Central Latin America' |
                location == 'Central Sub-Saharan Africa' |
                location == 'East Asia' |
                location == 'Eastern Europe' |
                location == 'Eastern Sub-Saharan Africa' |
                location == 'High-income Asia Pacific' |
                location == 'High-income North America' |
                location == 'North Africa and Middle East' |
                location == 'Oceania' |
                location == 'South Asia' |
                location == 'Southeast Asia' |
                location == 'Southern Latin America' |
                location == 'Southern Sub-Saharan Africa' |
                location == 'Tropical Latin America' |
                location == 'Western Europe' |
                location == 'Western Sub-Saharan Africa') %>%
   dplyr::select(,c(2,9:11))


IS_1990$val <- round(IS_1990$val,1)
IS_1990$lower <- round(IS_1990$lower,1)
IS_1990$upper <- round(IS_1990$upper,1)

IS_1990$'ASR   1990' <- paste(IS_1990$lower,IS_1990$upper,sep = '-')
IS_1990$'ASR   1990' <- paste(IS_1990$'ASR   1990',')',sep = '')
IS_1990$'ASR   1990' <- paste('(',IS_1990$'ASR   1990',sep = '')
IS_1990$'ASR   1990' <- paste(IS_1990$val,IS_1990$'ASR   1990',sep = ' ')
YLDs_ASR_1990 <- IS_1990[,c(1,5)]
head(YLDs_ASR_1990)

## 2021
IS_2021 <- IS %>%
   filter(year ==2021 &
                age =='Age-standardized' &
                metric == 'Rate' &
                measure   =='YLDs (Years Lived with Disability)' &
```

```
                  sex =="Both" &
                  rei=="Spinal Injuries"
    ) %>%

    filter(   location == 'Andean Latin America' |
                      location == 'Australasia' |
                      location == 'Caribbean' |
                      location == 'Central Asia' |
                      location == 'Central Europe' |
                      location == 'Central Latin America' |
                      location == 'Central Sub-Saharan Africa' |
                      location == 'East Asia' |
                      location == 'Eastern Europe' |
                      location == 'Eastern Sub-Saharan Africa' |
                      location == 'High-income Asia Pacific' |
                      location == 'High-income North America' |
                      location == 'North Africa and Middle East' |
                      location == 'Oceania' |
                      location == 'South Asia' |
                      location == 'Southeast Asia' |
                      location == 'Southern Latin America' |
                      location == 'Southern Sub-Saharan Africa' |
                      location == 'Tropical Latin America' |
                      location == 'Western Europe' |
                      location == 'Western Sub-Saharan Africa') %>%
    dplyr::select(,c(2,9:11))


IS_2021$val <- round(IS_2021$val,1)
IS_2021$lower <- round(IS_2021$lower,1)
IS_2021$upper <- round(IS_2021$upper,1)

IS_2021$'ASR   2021' <- paste(IS_2021$lower,IS_2021$upper,sep = '-')
IS_2021$'ASR   2021' <- paste(IS_2021$'ASR   2021',')',sep = '')
IS_2021$'ASR   2021' <- paste('(',IS_2021$'ASR   2021',sep = '')
IS_2021$'ASR   2021' <- paste(IS_2021$val,IS_2021$'ASR   2021',sep = ' ')
YLDs_ASR_2021 <- IS_2021[,c(1,5)]
head(YLDs_ASR_2021)


##### EAPC
YLDs_EAPC <- IS %>%

    filter(age =='Age-standardized' &
```

```
                 metric == 'Rate' &
                 measure =='YLDs (Years Lived with Disability)'&
                 sex =="Both" &
                 rei=="Spinal Injuries"
  ) %>%

  filter(    location == 'Andean Latin America' |
                   location == 'Australasia' |
                   location == 'Caribbean' |
                   location == 'Central Asia' |
                   location == 'Central Europe' |
                   location == 'Central Latin America' |
                   location == 'Central Sub-Saharan Africa' |
                   location == 'East Asia' |
                   location == 'Eastern Europe' |
                   location == 'Eastern Sub-Saharan Africa' |
                   location == 'High-income Asia Pacific' |
                   location == 'High-income North America' |
                   location == 'North Africa and Middle East' |
                   location == 'Oceania' |
                   location == 'South Asia' |
                   location == 'Southeast Asia' |
                   location == 'Southern Latin America' |
                   location == 'Southern Sub-Saharan Africa' |
                   location == 'Tropical Latin America' |
                   location == 'Western Europe' |
                   location == 'Western Sub-Saharan Africa') %>%
  dplyr::select(,c(2,7,9))

nations <- IS_1990$location
EAPC_YLDs                                                                                    <-
data.frame(location=nations,EAPC=rep(0,times=27),UCI=rep(0,times=27),LCI=rep(0,times=2
7))

for (i in 1:nrow(EAPC_YLDs)){
  nation <- as.character(EAPC_YLDs[i,1])
  a <- subset(YLDs_EAPC, YLDs_EAPC$location==nation)
  a$y <- log(a$val)
  mod_simp_reg<-lm(y~year,data=a)
  estimate <- (exp(summary(mod_simp_reg)[["coefficients"]][2,1])-1)*100
  low                  <-                  (exp(summary(mod_simp_reg)[["coefficients"]][2,1]-
1.96*summary(mod_simp_reg)[["coefficients"]][2,2])-1)*100
  high                                                                                         <-
(exp(summary(mod_simp_reg)[["coefficients"]][2,1]+1.96*summary(mod_simp_reg)[["coeffici
```

```
ents"]][2,2])-1)*100
    EAPC_YLDs[i,2] <- estimate
    EAPC_YLDs[i,4] <- low
    EAPC_YLDs[i,3] <- high
}


EAPC_YLDs$EAPC <- round(EAPC_YLDs$EAPC,2)
EAPC_YLDs$UCI <- round(EAPC_YLDs$UCI,2)
EAPC_YLDs$LCI <- round(EAPC_YLDs$LCI,2)

EAPC_YLDs$'EAPC_95%CI' <- paste(EAPC_YLDs$LCI,EAPC_YLDs$UCI,sep = ' to ')
EAPC_YLDs$'EAPC_95%CI' <- paste(EAPC_YLDs$'EAPC_95%CI',')',sep = '')
EAPC_YLDs$'EAPC_95%CI' <- paste('(',EAPC_YLDs$'EAPC_95%CI',sep = '')
EAPC_YLDs$'EAPC_95%CI' <- paste(EAPC_YLDs$EAPC,EAPC_YLDs$'EAPC_95%CI',sep = ' ')
EAPC_YLDs <- EAPC_YLDs[,c(1,5)]

YLDs <- YLDs_Num_1990 %>%
    left_join(YLDs_ASR_1990,by='location') %>%
    left_join(YLDs_Num_2021,by='location') %>%
    left_join(YLDs_ASR_2021,by='location') %>%
    left_join(EAPC_YLDs,by='location') %>%
    arrange(location)
View(YLDs)

# Supplementary Table 12-13 and Supplementary Figure 1-4 ---------------------------
---------------------

male_in<-read_excel("ARIMA 预测/SCI_ARIMA_global.xlsx",sheet=4)

male_in_ts <- ts(male_in$val, start = 1990, frequency = 1)

#  检查数据
plot(male_in_ts)

#  拟合 ARIMA 模型
fit1 <- auto.arima(male_in_ts)     ###自动拟合出最佳的 p,d,q 的值
fit1                               ###查看 p,d,q 的值
summary(fit1)                      ###查看模型的 AIC,BIC 的指标

#  检查残差，查看模型的拟合效果
checkresiduals(fit1)
residuals <- residuals(fit1)
acf(residuals, main="ACF of AirPassengers Data")
```

```
pacf(residuals, main="PACF of AirPassengers Data")
# Q-Q 图
qqnorm(fit1$residuals)
qqline(fit1$residuals)
# 美化 Q-Q 图
ggplot(data = data.frame(male_in_ts), aes(sample = male_in_ts)) +
  stat_qq() +
  stat_qq_line(col = "red") +
  ggtitle("QQ Plot of ARIMA Residuals") +
  theme_minimal()
# 进行预测
forecasted_values1 <- forecast(fit1, h = 15) ###预测未来 15 年数据
# 将预测结果转换为数据框
forecast_df1 <- data.frame(
  Year = c(time(male_in_ts), time(forecasted_values1$mean)),
  Value = c(as.numeric(male_in_ts), as.numeric(forecasted_values1$mean)),
  Type = c(rep("Actual", length(male_in_ts)), rep("Forecast",
length(forecasted_values1$mean)))
)
write.csv(forecast_df1,file = "male_ASIR_data.csv",quote = F,row.names = F)
# 绘制预测结果

p1<-ggplot() +
  geom_line(data = forecast_df1, aes(x = Year, y = Value, color = Type), size = 1.2) +
  geom_line(data = forecast_df1[forecast_df1$Type == "Forecast", ],
            aes(x = Year, y = Value, color = Type), size = 1.2) +
  geom_point(data = forecast_df1[forecast_df1$Type == "Forecast", ],
             aes(x = Year, y = Value, color = Type), size = 2, shape = 21,
             fill = "yellow", color = "black", stroke = 0.5) +
  geom_ribbon(data = data.frame(
    Year = time(forecasted_values1$mean),
    ymin = forecasted_values1$lower[,2],
    ymax = forecasted_values1$upper[,2]
  ), aes(x = Year, ymin = ymin, ymax = ymax), fill = "yellow", alpha = 0.2) +
  geom_vline(xintercept = 2021, linetype = "dashed", color = "grey", size = 1) +
  scale_color_manual(values = c("Actual" = "red", "Forecast" = "yellow")) +
  labs(title = "ASIR of Male", x = "Year", y = "ASIR") +
  ylim(0, 13) +
  theme_minimal() +
  theme(axis.line = element_line(color = "black"),
        plot.title = element_text(hjust = 0, vjust = 1, face = "bold", size = 14),
        plot.margin = margin(10, 10, 10, 10))
p1
```
